# Complex Residences and Sociality: How Coral Structure and Social Environment Influence Occupation Patterns in *Gobiodon* in Aquaria

**DOI:** 10.1002/ece3.71887

**Published:** 2025-07-28

**Authors:** Courtney A. Hildebrandt, Catheline Y. M. Froehlich, O. Selma Klanten, Marian Y. L. Wong

**Affiliations:** ^1^ Centre for Sustainable Ecosystems Solutions, School of Earth, Atmospheric and Life Sciences University of Wollongong Wollongong New South Wales Australia; ^2^ Department of Biology University of Alabama Tuscaloosa Alabama USA; ^3^ Dauphin Island Sea Lab Dauphin Island Alabama USA; ^4^ School of Life Sciences University of Technology Sydney Sydney New South Wales Australia

**Keywords:** animal sociality, coral gobies, coral reef, *Gobiodon*, habitat complexity, habitat size, social status

## Abstract

Habitat size and complexity are two of many important characteristics that can have profound effects on individual survival, growth, and reproduction. However, little is known about the influence these characteristics may have on sociality and how this may be altered in response to environmental stress. Among marine fishes, coral‐dwelling gobies are highly specialized cryptobenthic fish that live almost exclusively between the branches of *Acropora* corals, on which they rely for survival and reproduction. The study investigated whether grouping patterns and habitat choice decisions of a facultatively social species, *Gobiodon quinquestrigatus* , were influenced by habitat complexity and size. Coral structures were 3D printed with eco‐friendly polymers to create high and low complexity *Acropora* structures, allowing coral complexity and size to be precisely manipulated. Replicated trials consisted of three sequential 24‐h treatments: (1) breeder pair only, (2) breeder pair with the addition of a subordinate, and (3) breeder pair and subordinate with a reduced size of the high complexity coral structure. All individuals were observed more frequently in the high complexity coral structure. Females chose the high complexity structure most frequently, with males and subordinates less likely to choose the high complexity structure. Breeders were more likely to occupy the high complexity coral structure even when the size of the high complexity coral structure was reduced. Breeders were observed switching more frequently in their first round (breeder pair only) compared to the latter rounds when the subordinate was added and when the coral size was reduced. Breeder males switched marginally more than breeder females. However, subordinates performed the most switches consistently throughout the two rounds they were present. These results highlight the dynamic relationship between sociality and structural complexity in a habitat specialist reef fish, with implications for our understanding of social maintenance in response to climate change and habitat loss.

## Graphical abstract

Using the novel methodology of 3D printing, we precisely and accurately manipulated complexity to observe habitat choice behavior in coral‐dwelling gobies. The social status and complexity of the coral structure influenced the choice of the individuals.

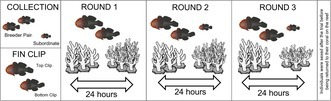

## 1 Introduction

Habitat characteristics can have profound impacts on the fitness of organisms that occupy the environment. Habitat size is known to influence the abundance and diversity of many vertebrate and invertebrate taxa (Ricklefs and Lovette 1999; Reed and Hovel 2006; Schuler et al. 2017; Lawrence et al. 2018). For example, Ricklefs and Lovette (1999) reported a significant positive correlation between island habitat area and the species richness observed in birds, bats, butterflies, and reptiles. For species that form groups, habitat size can also impose constraints on group size and composition (Furness and Birkhead 1984; Gerard and Loisel 1995; Janson and Goldsmith 1995; Bonabeau et al. 1999; Gillespie and Chapman 2001; Atwood 2006). For example, a group of coyotes is typically larger in forest patches with greater areas (Atwood 2006). These habitat size constraints on group size are likely to be more profound for site‐attached organisms that are confined to living in discrete patches, because of the close association between habitat size and food, shelter, and breeding site availability (Clutton‐Brock et al. 2008; Chapin 2014).

In tropical marine environments, corals are the primary building block of ecosystems, and coral size is well known to influence fish assemblage structure (Schroeder 1987; Acosta and Robertson 2002; Holbrook et al. 2002; Jordan et al. 2005; Feary et al. 2007; Noonan et al. 2012). Variation in potential living space accounts for more than half of the variation in species richness and total abundance in the fish assemblages (Holbrook et al. 2002). However, other habitat metrics such as coral complexity have been observed to influence fish abundance and species richness (Gratwicke and Speight 2005; Coker et al. 2012; Komyakova et al. 2013; Emslie et al. 2014; Darling et al. 2017). For example, reductions in coral complexity can result in greater changes to fish assemblages than loss of live coral cover alone (Emslie et al. 2014). This suggests that coral size as well as complexity can have a profound effect on fish assemblages at the ecosystem level.

In addition, coral characteristics can also affect individual fish species, particularly those that rely on the corals for most if not all aspects of their survival and reproduction (Dirnwöber and Herler 2007; Untersteggaber et al. 2014). For such species, it has been long established that coral size influences group size (Wong et al. 2005; Thompson et al. 2007; Schiemer et al. 2009). Additionally, coral size is typically correlated with body size ratios, mating systems, and sex allocation in coral dwelling fishes (Wong et al. 2005, 2012; Thompson et al. 2007; Wong 2010; Rueger et al. 2021). For example, subordinate coral gobies (*Paragobiodon xanthosoma*) choose to form groups in larger corals over pairs in smaller corals as the size was increased, therefore trading off immediate reproduction for access to a larger habitat (Wong 2010). However, it is currently less well known whether other coral complexity can influence sociality to the same extent. In addition, it is not clear whether individuals that differ in sex, size, or status respond similarly to changes in coral size and complexity. Therefore, there is still a significant knowledge gap pertaining to how coral size and complexity interact to determine observed sociality and coral habitat occupancy.

Coral dwelling gobies from the genus *Gobiodon* are a useful model system for investigating the relationship between habitat and social variables. *Gobiodon* species are coral dwelling fishes that are obligately associated with certain host corals; however, despite being highly site attached, they can move across relatively large distances in order to find favorable habitat (Froehlich et al. 2022). *Gobiodon* also display a wide range of social phenotypes, including pair and group forming (Hing et al. 2018; Froehlich et al. 2024). Within groups, reproduction is dominated by a large breeding pair, with smaller non‐breeding subordinates forming a size‐based hierarchy where individuals queue for breeding access (Munday 2000; Hing et al. 2018; Froehlich et al. 2024). These subordinate individuals are subject to the threat of conflict or even forcible eviction by the dominant breeder pair (Wong et al. 2007). Coral size has been demonstrated to provide a number of benefits to *Gobiodon* species, with individuals demonstrating increased growth on larger coral colonies (Pereira and Munday 2016). It has previously been demonstrated that coral complexity can influence goby morphology, suggesting that there is a pre‐existing relationship between coral complexity and their biology (Untersteggaber et al. 2014; Wehrberger and Herler 2014). However, there have been no studies investigating the role of coral complexity, habitat choice, and grouping decisions.

Here we used an innovative method of three‐dimensional (3D) printing to generate corals of high and low complexity to precisely tease apart the influence of coral complexity, size, and sex/social status on the habitat choice of coral dwelling gobies ( *Gobiodon quinquestrigatus* ). The goal of the study was to mimic the social environment that *Gobiodon* would be subjected to in situ on the reef when deciding on coral occupation. Therefore, decisions were made in the presence of a partner (for established pairs) or in the presence of other individuals (for subordinate individuals). Gobies were provided with a choice of low and high complexity as well as differences in coral size. Each trial has a breeder pair and a non‐breeding subordinate present to look at the difference between sexual/social status. It was predicted that individual coral complexity, coral size, and sexual/social status would influence the choice of coral structure and frequency of movement between the choice options or switches. More specifically, it was expected that all individuals would choose corals of higher complexity and larger size, with breeders displaying a stronger choice for high complexity than subordinates. It was also predicted that subordinate individuals would switch between coral structures more often compared to breeders. Finally, a reduction in coral size was expected to result in reduced occupation of the high complexity coral.

## 2 Methods

### 2.1 Site Description & Study Species

Field collections of coral gobies and coral choice trials were conducted from the 5th of March to the 30th of April of 2022 at One Tree Island (OTI) Research Station on the southern Great Barrier Reef, Australia (23°30′ 21.66″S, 152°5′ 27.54″E). *Gobiodon quinquestrigatus* was chosen for the study as it is widespread and abundant throughout the One Tree Island (OTI) reef (Hildebrandt, pers. obs.). *Gobiodon quinquestrigatus* is also a coral host generalist, being found occupying a range of *Acropora* corals (Munday et al. 1999; Froehlich et al. 2022) and is considered facultatively social, with a sociality index close to 0.5 in some locations (Froehlich et al. 2024). A sociality index value of 0.5 means that individuals will frequently occupy corals as a breeding pair but also have the capacity to form social groups with subordinate individuals occupying the same coral as a breeding pair. Commonly observed host species include *A. cerealis, A. humilis, A. millepora, A. nasuta, A. tenuis*, and *A. valida* (Munday et al. 1999), with *A. humilis* being the most commonly inhabited coral at OTI (Hildebrandt & Froehlich, pers. obs.). Therefore, *A. humilis* was chosen for replication via 3D printing.

### 2.2 Designing and Printing 3D Coral Structures

The design of our 3D coral structures was based on data collected from photographs of 29 colonies of *Acropora humilis* at One Tree Island between the 28th of January and the 19th of February 2019. Corals were photographed using an Olympus Tough OM System Camera. Only corals that were inhabited by gobies were photographed to ensure that coral structure was appropriate for habitation.

**FIGURE 1:**
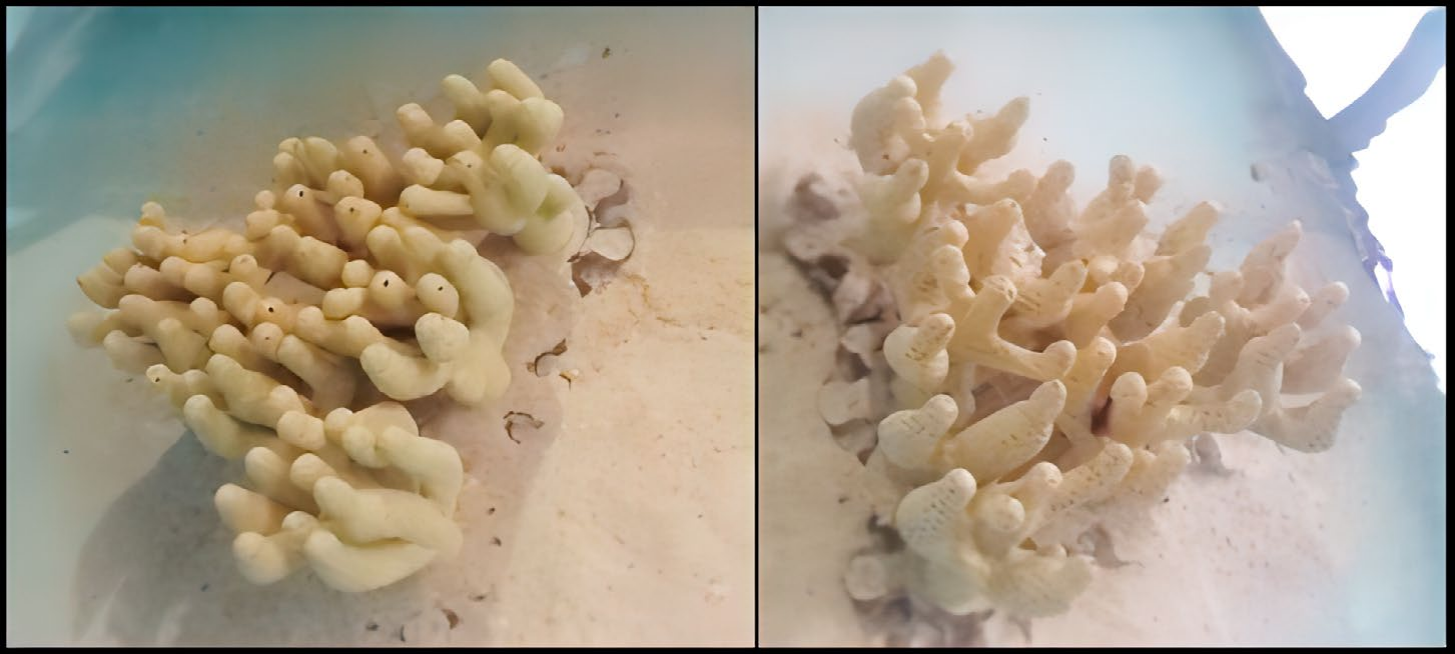
Image of the 3D printed coral structures in the trial tank. High complexity coral structure is on the left, while the low complexity coral structure is on the right.

In order to quantify coral complexity for our 3D printed corals, key coral dimensions were extracted from photographs using Image J (Schneider et al. 2012). To do this, the image was first scaled to reflect the coral dimensions (maximum length, width and depth) measured in the field. Following that, each of the following measurements was extracted to one decimal place on the scaled imported image: coral diameter (cm), number of branches, surface area (cm^2^), and average inter‐tip distance between branches (cm). These coral characteristics were previously used to quantify coral complexity and architecture (Untersteggaber et al. 2014; Wehrberger and Herler 2014). These complexity measurements were then plotted to estimate the lowest and highest complexity values of inhabited corals (Figures S1 and S2). Based on these values, one low complexity and one high complexity 3D printed coral model was made to accurately represent a low and a high complexity coral of the species seen in situ (Table S1). Both coral designs produced a coral of equal diameter and height with the same branch widths. The main distinguishing characteristic between the high and low complexity designs was the interbranch distance (IBD), with the high complexity design resulting in an average of 1.5 cm between branches, while the low complexity design had an average of 3.2 cm between branches. This variation resulted in the high complexity coral having a higher branch density than the low complexity coral, with a greater number of branches closer together in the same overall dimensions as the low complexity coral (Figure 1).

**FIGURE 2:**
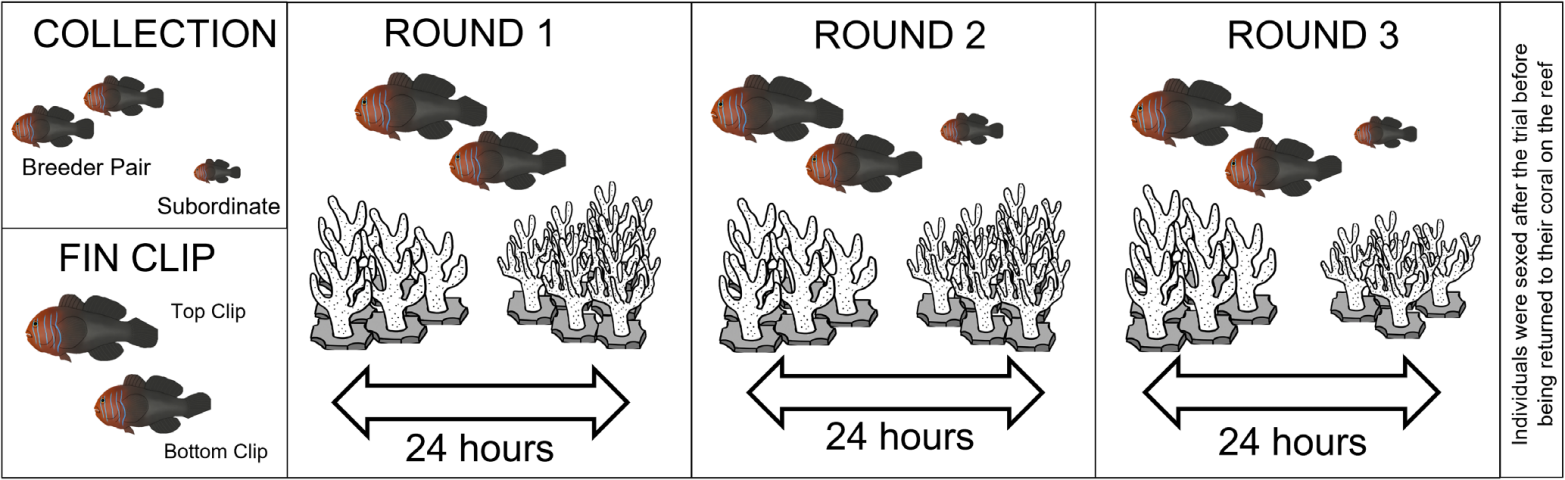
Diagram showing the overall experimental procedure, pre‐trial procedure, and the three trial rounds with the individuals present (large = breeder pair, smaller = subordinate non‐breeder), coral structure (left = low complexity coral, right = high complexity coral) and the length of time of each round (24 h). The location of the coral structures at either end of the tank was randomized for each new experiment trial. Note that the high and low complexity corals are comprised of six subunits, two of which were removed from the high complexity coral in round 3 to simulate a reduction in coral size.

To enable manipulations of both size and complexity, six identical interlocking subunits of the high and low complexity coral structures were printed. The interlocking aspect was important as it enabled us to remove subunits for the experimental coral size reductions (see below Figures 1 and 2). All 3D models were designed and printed by the Translational Research Initiative for Cellular Engineering and Printing (TRICEP) using medical grade polylactide (PLA), a non‐toxic and ecologically safe material. The designs were based on the images collected of *Acropora humilis* on the 2019 field trip and designed to meet the measurements provided for the high and low complexity variations calculated. Due to the requirements of the design to be modular for size reduction, limitations of 3D printing and transportation restrictions, some slight variations from the appearance of *Acropora humilis* are visible. However, the structure and arrangement of the branches were made to replicate the measurements taken from corals in situ.

The printing materials had previously undergone toxicology screenings conducted by Envirolab Services Pty. Ltd. (https://www.envirolab.com.au/) to ensure that they would not harm the fish in the trial. Once printed, the models were thoroughly washed and dried before being coated in an aquarium safe non‐toxic sealant in a light sandstone color. The color treatment gave the printed structures a more realistic appearance and covered any minor blemishes or differences that may have occurred during the printing. After the sealant was dry, the models were given another thorough clean and multiple rinses to ensure there was no remaining residue on the structures before the start of the experiment.

### 2.3 Goby Collection

*Gobiodon quinquestrigatus* were collected by snorkelers or on SCUBA (Munday et al. 1999; Froehlich et al. 2023). Once located, individuals were collected by spraying a dilute solution of Aqui‐S (1:10 Aqui‐S: seawater) over the coral to lightly anaesthetize them, followed by wafting a current of water by hand to gently remove them from the coral (Munday and Wilson 1997). The coral was then marked using a Garmin eTrex 10 GPS console to enable the return of the individuals to the original coral at the conclusion of the trials. Established breeder pairs were collected from the same coral to avoid any behavioral confounds created by unfamiliar partners, and the subordinate was collected from a separate coral to simulate an unfamiliar subordinate entering the coral (Wong 2010; Froehlich et al. 2022). An individual was classified as a subordinate if it was less than 20 mm in standard length and observed occupying a coral in isolation.

Once collected from the coral, all individuals were transferred to a sealed zip lock bag containing seawater and placed in a large container of regularly refreshed seawater on the boat to maintain constant temperature and aeration. Within 30 min of collection, individuals were transported back to the research station and transferred to holding tanks within the wet laboratory areas. Fish were housed in glass tanks (50 × 30 × 30 cm) on‐site overnight for acclimation before entering the trial tank. Holding tanks were equipped with seawater being constantly pumped from the surrounding lagoon, allowing conditions in the tanks to mimic the natural habitat. The breeder pair was housed in a holding tank together to maintain the pre‐existing familiarity, whereas the subordinate individual was housed alone in a separate tank. All fish were provided with coral rubble for shelter within holding tanks. Additionally, the wet laboratory area is located under a shelter to ensure that changes in weather would not exert changes on the environment of the holding tank or trial tank throughout the course of holding or experimentation. No food was added because water was pumped directly from the ocean and therefore would provide planktonic food for the gobies.

Before experimental trials, breeder individuals were lightly sedated using Aqui‐S solution, and a small fin clip on the caudal tail was taken (~1–2 mm) from the upper portion of the caudal fin on one partner and the lower portion of the other partner to enable visual identification within the trial tank. Elastomer tagging was attempted using fluorescent elastomer (Northwest Marine Technologies Inc.), but the tags were usually not visible against their dark bodies without handling the fish. After fin clipping, individuals were given at least 12 h to recover before the trials commenced. Subordinates were not fin clipped, as they were easily identifiable based on their smaller relative body size.

### 2.4 Trial Methodology

To investigate the effects of coral size and complexity on habitat decisions, the experiment was performed over 3 consecutive trial rounds: the first involved only the breeder male and female, the second round involved the breeding pair plus subordinate non‐breeder, and the third round involved all individuals with the size of the high complexity coral reduced by removing 2 of the 6 subunits of the high complexity coral structure (Figure 2). Each of these trial rounds lasted for 24 h from 8 am to 8 am the next day, ensuring that all rounds had equal day and night periods.

**FIGURE 3:**
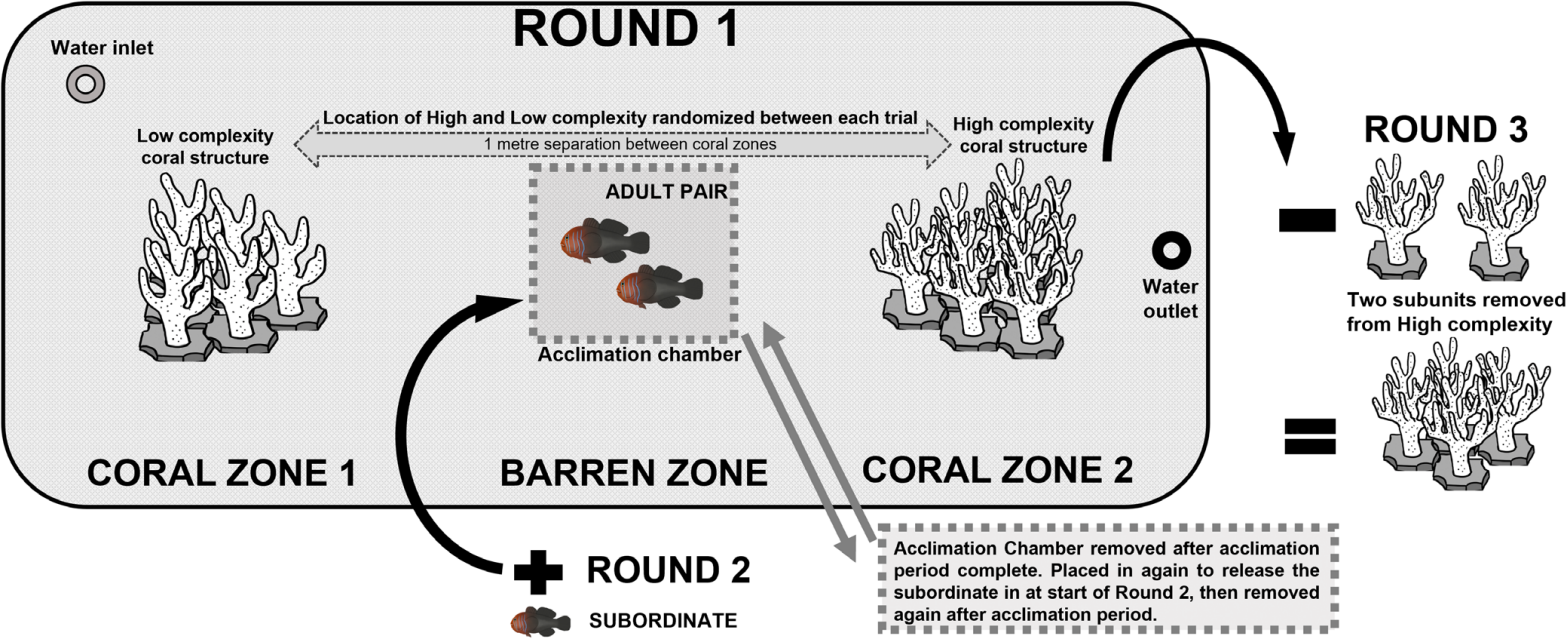
Trial tank design showing the experimental procedure and tank environment during the three trial rounds. The tank had constant water flow through as seen by the water inlet and outlet, with three zones: (1) coral zone 1, (2) barren zone and (3) coral zone 2. The location of the high and low complexity coral structures was randomized between coral zone 1 and 2 at the start of each trial. A pair of *Gobiodon quinquestrigatus* were added to the acclimation chamber at the start of round 1 and a solitary juvenile of the species at the start of round 2. At the beginning of trial round 3, two subunits were removed from the periphery of the high complexity coral to mimic a reduction in coral size.

Each experimental trial was conducted in a trial tank (100 × 200 × 50 cm) that was divided into three distinct zones (Figure 3). The two coral structures (high and low complexity) were randomly placed at either end of the trial tank, and in the middle of the tank, a ‘barren zone’ was established where no structure was provided, separating the two coral structures by 1 m (Figure 3). At the start of each trial, an acclimation chamber was placed in the middle of the barren zone (Figure 3). The acclimation chamber consisted of a clear acrylic box (20 × 20 × 30 cm) with small rectangle cutouts in two of the sides, covered in mesh, to allow the flow through of water as well as chemical and visual cues. The top of the chamber had a circle cut out, which allowed the fish to be gently placed into the acclimation chamber from above. The bottom of the chamber was open to allow the fish to sit on the trial tank bottom and be minimally disrupted when the chamber was removed at the conclusion of the acclimation period.

To begin trial round 1, the breeder pair were gently transferred from their holding tank and placed into the acclimation chamber in the barren zone for 10 min for acclimation to the trial tank. Once this period was complete, the acclimation chamber was gently removed, and the pair were able to move freely within the experimental tank. The location of each partner was recorded at multiple time points: immediately, after 10 min, after 1 h, after 12 h and after 24 h, from the commencement of the trial round. During the trial period the area around the tank was kept clear of passing people and movement to reduce any external disturbances. The location of each individual was recorded as being in the high or low complexity corals or scored as ‘no choice’ if they were not residing in either structure.

After 24 h, trial round 2 commenced by placing the acclimation chamber in the barren zone and transferring the trial subordinate into the chamber for a 10‐min acclimation period. At the end of this period, the acclimation chamber was removed, and the subordinate was allowed to move freely within the experimental tank. The location of all three individuals was recorded at each of the time points listed previously. After a further 24 h, trial round 3 commenced by removing two of the six subunits (controlled decrease in size) from the periphery of the high complexity coral structure, thereby decreasing the overall size of the coral by 33%. This was done to mimic previously recorded decreases of approximately 30% coral size resulting from climatic disturbances (Froehlich et al. 2021). The change was conducted gently while the gobies were still in their chosen coral to keep disturbance to a minimum. Two subunits of the low complexity were also slightly moved; however, they remained part of the low complexity coral structure to control for disturbance without changing the size of the low complexity structure. The location of all three individuals was then recorded at the previously listed time points over the next 24 h.

After the completion of the trials, the fish were gently removed from the trial tank using hand nets and placed into small holding tanks. Each fish was then individually sedated using the Aqui‐S mixture at a dilution ratio of 1:10 with seawater. Standard length, total length (mm ±0.1 mm) and the sex of the breeder pair individuals were measured. Length measurements were recorded using calipers, and the sex of the breeder pair was determined using an observation microscope (following Wong et al. 2007). The subordinate was not sexed as they are assumed to be immature individuals (Munday et al. 1998). The sexing and measuring of individuals occurred only after the completion of the trials to minimize any physical handling effects on fish prior to the trials. Once the measuring and sexing were completed, individuals were placed into the holding tanks to recover from the anesthetic before being returned to their original host coral on the reef. A 100% survival rate was recorded from this process, with all fish returned to their original host coral on the reef in good health.

A total of 16 trials were conducted with 3 individuals in each (1 breeder male, 1 breeder female and 1 subordinate individual). Therefore, 48 individuals were observed, with a total of 256 location observations recorded over the course of the study.

## 3 Statistical Analysis

All analyses were conducted in R (v4.2.1) (R Core Team 2021), using the packages ‘mclogit’ (Elff 2022), ‘nnet’ (Venables et al. 2002), ‘memisc’ (Elff 2023), ‘sJPlot’ (Lüdecke 2023), ‘lme4’ (Bates et al. 2015), ‘lmertest’ (Kuznetsova et al. 2017) and ‘car’ (Fox and Weisberg 2019).

To examine whether the high complexity coral structure was chosen significantly more often than the low complexity structure and no‐choice options, a chi‐squared goodness of fit test was performed. All recordings 12 h post the addition of the individuals were included in the analysis as it was at this point that the gobies appeared to settle within the trial tank.

To examine the factors influencing host coral choice, two multinomial generalized linear mixed models were run. The first model examined the effect of sexual/social status on the choice of coral host, with group ID as a random factor. Note that the variable “sex” encompassed 3 categories, that is, breeder male, breeder female, and subordinate non‐breeder, and therefore reflects a combination of sex and status. This first model was run twice, with (1) breeder females as the reference category and (2) breeder males as the reference category, thus ensuring that comparisons between all present sexes could be observed. Only the choices made after the subordinate was added were used in the analyses, as this ensured all individuals were present at all time points used in the analysis. The second model examined the effect of trial round on host choice decisions, with group ID as a random effect. Each of the three trial rounds was represented by the recorded locations at 12 and 24 h post commencement. The model was run in two stages: (1) the breeder pair only across all trial rounds and (2) subordinates only across the second and third trial rounds. This division accommodated for the fact that the subordinates were not present during the first trial round while also enabling comparisons between breeder and subordinate decisions to be examined. The location 12 h post addition was used as the reference category for both status groups respectively (breeder and subordinate), as there were very few choices made during the initial observation recorded immediately after addition.

To examine the factors influencing the number of switches, generalized linear mixed models were used with sexual/social status, trial round, and their interaction as predictors of the number of switches performed (response). The analysis was conducted in three stages: (1) breeder male and female only and during all rounds, and (2) breeder male, female, and subordinate during the second and third trial rounds, and (3) subordinate during the second and third rounds (however due to overfitting, a generalized linear model without a random factor was performed due to overfitting). This design was used due to the subordinates not being present during all rounds of the trial. A random factor of group identification was included in both analyses. The ‘Poisson’ distribution family was used in both models as the number of switches is a count variable. A negative binomial distribution was initially attempted, but the Poisson model was deemed a better fit to the data based on AIC values. The models were checked for overdispersion, homogeneity, and collinearity.

## 4 Results

A total of 48 individuals were observed in 16 trials, with each trial consisting of 3 rounds with multiple location observations. Therefore, a total of 256 location observations were recorded over the course of the experiment. Overall, a significantly greater percentage of occupation observations of the fish (70%) were in the high complexity coral regardless of sexual/social status or trial round (*χ* ^2^ = 162.2, df = 2, *p* < 0.001).

**FIGURE 4:**
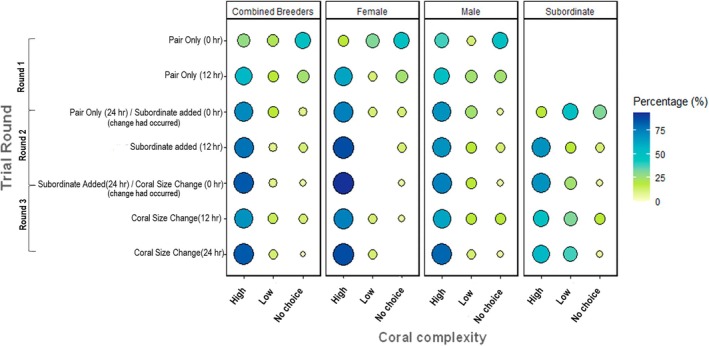
Balloon plot showing proportion of *Gobiodon quinquestrigatus* individuals in each coral type at each time point throughout the study. Time points for each round are shown on the *y*‐axis and coral complexity choices shown on the *x*‐axis. The observed choices were the high complexity coral, low complexity coral or neither coral, which was recorded as a “No choice” choice. The female, male and subordinate categories each include 16 individuals (*n* = 16) per group with the combined breeder category composed of the breeder males and breeder females combined (*n* = 32). The color and size of the circles represent the percentage of individuals of that status category observed displaying the choice at the end of each recording period.

Coral choice decisions varied depending on sexual/social status. Specifically, breeder females were observed choosing the high complexity coral structure more often than breeder males and subordinates (Figure 4 and Table 1). The high complexity preference was greater during the addition of the subordinate (round 2) and following the reduction in size of the high complexity coral (round 3) (Figure 4 and Table 1). Males were observed in the high complexity coral structures slightly more often than subordinates (Figure 3); however, the difference between males and subordinates was not significant (Table 1). In addition, coral choice decisions for breeders varied depending on the trial round. Specifically, breeders were more likely to be observed in the high complexity coral compared to not residing in any coral 24 h after the commencement of round 2 and round 3 than during the first round (reference category) (Figure 4 and Table 2). There were no other significant differences in preferences between the trial rounds. Breeders were observed consistently within the high complexity coral structure throughout the experiment, with only small increases in occupation of the low complexity and no‐choice options after the introduction of the subordinate and the reduction in coral size.

**TABLE 1:** Results of the multinomial generalized linear mixed model for the effects of individual status on the choice of host coral structure.

Predictor		Low vs. high			No choice vs. high			Low vs. no choice		
		OR	CI	*p*	OR	CI	*p*	OR	CI	*p*
Status (female reference)	Female	Reference category			Reference category			Reference category		
	Male	3.69	1.05–13.00	**0.041**	1.87	0.53–6.62	0.333	2.00	0.39–10.24	0.405
	Subordinate	7.80	2.31–26.29	**0.001**	2.37	0.66–8.52	0.183	3.33	0.68–16.37	0.138
Status (male reference)	Male	Reference category			Reference category			Reference category		
	Female	0.27	0.08–0.95	**0.041**	0.54	0.15–1.90	0.333	0.50	0.10–2.57	0.405
	Subordinate	2.11	0.84–5.32	0.112	1.27	0.39–4.17	0.689	1.66	0.45–6.12	0.442

*Note:* Trial group identification was included as a random factor. Bold text and darker cell shading denote significance (*α* = 0.95).

Abbreviations: CI, confidence interval; OR, odds ratio; *p*, significance value.

**TABLE 2:** Results of the multinomial generalized linear model for the effects of individual sexual/social status on the choice of host coral structure.

Predictor		High vs. no choice			Low vs. no choice			Low vs. high		
Trial rounds		OR	CI	*p*	OR	CI	*p*	OR	CI	*p*
Breeder male and female model	Pair +12HR	Reference category			Reference category			Reference category		
	Pair +24HR/subordinate added	4.06	0.85–19.35	0.078	2.55	0.41–15.69	0.312	0.66	0.14–3.04	0.591
	Subordinate added +12HR	3.64	0.85–15.57	0.081	0.86	0.13–5.77	0.873	0.24	0.04–1.38	0.110
	Subordinate added +24HR/coral change	8.43	1.45–48.88	**0.017**	1.69	0.20–14.49	0.630	0.21	0.04–1.20	0.079
	Coral size change +12HR	2.27	0.57–9.07	0.246	1.24	0.23–6.87	0.801	0.57	0.12–2.73	0.476
	Coral size change +24HR	16.75	1.78–157.72	**0.013**	4.65	0.38–56.33	0.226	0.30	0.06–1.52	0.144
Subordinate model	Subordinate added +12HR	Reference category			Reference category			Reference category		
	Subordinate added +24HR/coral change	1.99	0.15–26.86	0.599	2.68	0.15–46.87	0.496	1.33	0.23–7.64	0.745
	Coral size change +12HR	0.45	0.06–3.59	0.450	1.13	0.11–11.50	0.918	2.34	0.41–13.17	0.331
	Coral size change +24HR	1.56	0.11–21.28	0.737	4.06	0.25–67.21	0.322	2.47	0.46–13.20	0.284

*Note:* Trial group identification was included as a random factor. Bold text and darker cell shading denote significance (*α* = 0.95).

Abbreviations: C, confidence interval; OR, odds ratio; *p*, significance value.

When considering subordinates only, subordinate choices did not significantly differ between any trial round compared to their reference round; hence, their choices were not influenced by trial round (Figure 4 and Table 2). A small decrease in the occupation of the high complexity was observed as the rounds progressed, with a corresponding increase in the occupation of the low complexity coral structure (Figure 4). Additionally, a small increase in the number of no‐choice options occurred 12 h after the reduction in size of the high complexity coral.

**FIGURE 5:**
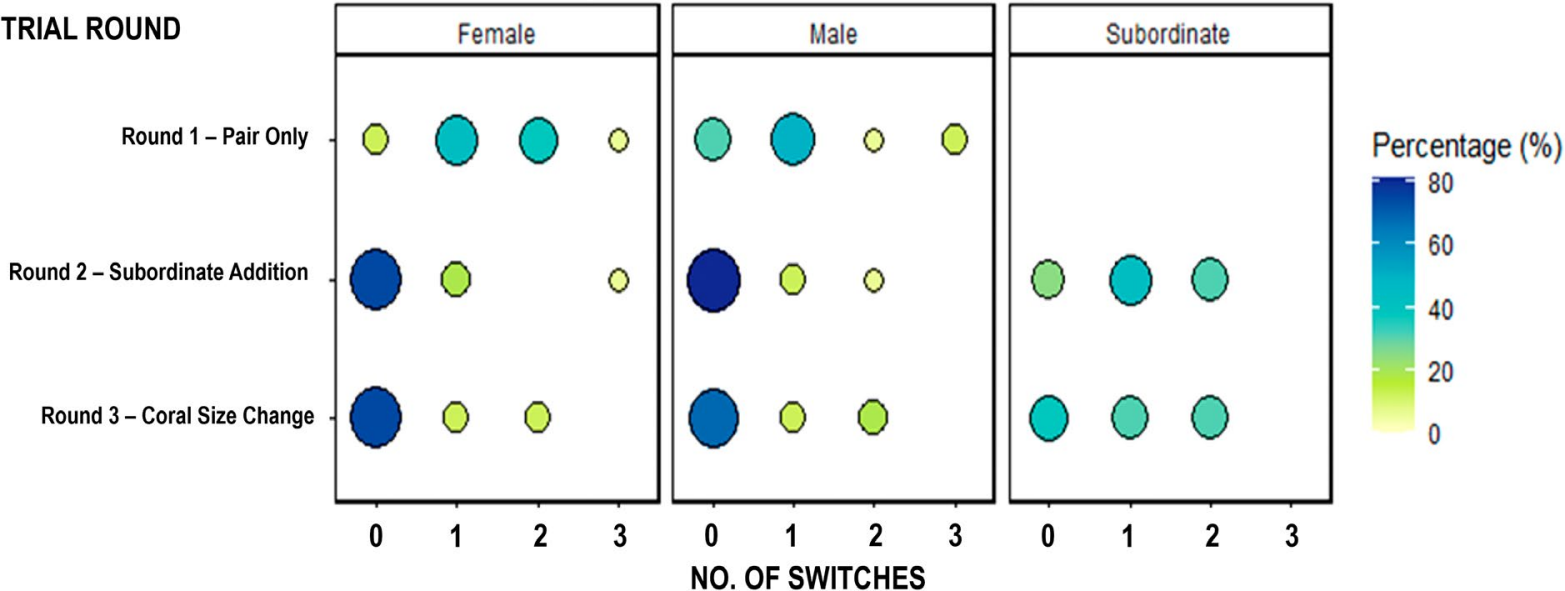
The proportion of each of the observed number of switches that could be performed by individuals of each sexual/social status (female, male and subordinate) during each of the three rounds (Pair only, Subordinate added and Coral size change). The size and color of the circle represent the proportion of individuals in that sexual/social status category at that trial round that performed that number of switches.

On average, individuals switched between corals 0.73 ± 0.86 times, with individuals switching between zero and three times within a 24‐h round (Figure 5). The number of switches performed by breeder males and females across all rounds did not differ significantly (Table 3). However, after the subordinate was added (i.e., trial round 2), the number of switches performed by breeders reduced (Table 3). Females were observed switching more than males in the first round (Figure 5). Both breeder males and females displayed a higher frequency of switches in trial round 1 than compared to trial round 2 and 3, as overall reduction in switching was observed during trial rounds 2 and 3 (Figure 5 and Table 3). Subordinates did not decrease their level of switching during trial round 3 compared to trial round 2, hence there was no correlation with the trial round and the number of switches performed by subordinates (unlike for breeders) (Figure 5 and Table 3).

**TABLE 3:** Results of the generalized linear mixed model for the effect of sexual/social status and trial round on the number of switches performed by an individual.

Model	Variable	Analysis of deviance (Wald *χ* ^2^)	Variable degrees of freedom	Residual degrees of freedom	*p*
Breeder male and females only (rounds 1–3)	Sexual/social status	0.939	1	89	0.332
	Round	13.108	2	89	**0.00143**
	Sexual/social status*round	1.046	2	89	0.593
Breeder male, females and subordinates (rounds 2 and 3)	Sexual/social status	9.563	2	89	**0.00838**
	Round	< 0.001	1	89	0.999
	Sexual/social status*round	1.360	2	89	0.507
Subordinate only (rounds 2 and 3)	Round	0.125	1	29	0.7238

*Note:* Trial group identification was included as a random factor. Bold text and darker cell shading denote significance (*α* = 0.95).

## 5 Discussion

Habitat characteristics are known to play an important role in governing individual group living decisions, particularly for species that are highly specialized and rely on their habitat for food, shelter, and reproduction. Here we found that habitat complexity was a key driver of habitat choice decisions, and this effect was modulated depending on the sexual/social status of an individual and both the physical and social environment present in the trial. Overall, individuals expressed a stronger affinity for the high complexity coral structure compared to the lower complexity structure, and this affinity was greatest in breeder females. Changing coral sizes did not alter the preference for high complexity coral structures seen in breeder individuals. However, during the first 24 h, both breeder males and females did explore the trial tank and switch between the high and low complexity structures more than the following 48 h. Subordinates were observed switching consistently between the high and low complexity structures regardless of the time period. These findings suggest that a higher complexity habitat is important for *Gobiodon* and a reduction in coral size does correlate with individuals changing corals. However, it is apparent that social status may influence access to the high complexity habitat. These results underline the need to preserve complex coral habitats, which are diminishing as live coral coverage is diminishing (Mills et al. 2023).

The sex and social status of individuals modulated habitat choices in relation to complexity, with breeder females occupying the high complexity coral structure significantly more than breeder males and subordinates, suggesting that breeder females have a stronger preference for high complexity structures. One possible explanation is that higher complexity corals provide a greater source of food, which is necessary for females as producing gametes is energetically costly and food is usually limited (Wong et al. 2008). *Gobiodon* have been reported to consume small invertebrates, filamentous algae, and occasionally coral polyps (Brooker et al. 2010); thus, increased host structural complexity could provide more surface area for algae and other invertebrate prey to grow (Brooker et al. 2010; González‐Rivero et al. 2017). Additionally, higher structural complexity is often associated with reduced predation and competition, therefore improving chances of both survival and reproduction (Sale et al. 1984; Holbrook et al. 2000; Beese et al. 2023). Increased food availability and shelter in more complex habitats may therefore enhance the breeder female's survival and reproductive success.

In comparison, there was no significant difference between the choices of breeder males and subordinates when compared to breeder females. However, while the males appeared to exhibit similar occupation decisions to the subordinates, the mechanisms driving these behaviors could differ. In comparison to females, male reproduction is less energetically costly, producing sperm in comparison to eggs (Bateman 1948; Parker 1970, 1982; Kokko and Jennions 2003, 2008). The lower energetic requirements could lead to male breeders having a reduced bias to the higher resource availability in the high complexity in contrast to breeder females. While paternal care is exhibited by other species of monogamous coral gobies (Whiteman and Côté 2004; Wong et al. 2005; Hernaman and Munday 2007), there were no eggs laid during the trials conducted; therefore, male breeders did not exhibit energetically costly paternal care. With breeder males not required to exhibit paternal care, more energy may have been invested in nest and territory defense (Goiran and Shine 2015), as aggression and defense in breeder individuals may have been triggered by the subordinate attempting to enter the coral (Wong et al. 2008). With breeder males having more energy availability due to the lack of paternal care, breeder males may have been more inclined to invest in defense, possibly even moving around the tank to exhibit aggression towards the subordinate. These lower energy requirements of the breeder males could therefore explain the movement of the breeder males throughout the trial tank beyond the initial exploratory period shared with the breeder female.

Subordinates, on the other hand, are more likely to be influenced by the actions of the breeders. Subordinates generally face increased susceptibility to predation due to their smaller size (Buston 2003; Chan et al. 2019); therefore, subordinates may be expected to choose the higher complexity structure over the lower complexity structure. However, the subordinate individuals could have been evicted from or prevented from joining the high complexity coral structure by the breeding pair, forcing them to take up residence in the low complexity coral structure or occupy the outskirts of the trial tank. Therefore, the presence of the breeding pair may have prevented subordinates from obtaining their desired host coral choice, at least within the timeframe of our experiment. Over longer time periods, subordinates may be accepted, and a stable social queue for breeding may form, which has been observed in the closely related *Paragobiodon* (Wong et al. 2007). Therefore, the presence of the breeder pair within the trial tank environment was a likely strong contributor to the observed coral occupation choices observed in the subordinate individuals.

When comparing across trial rounds, both male and female breeders were significantly more likely to be observed in the high complexity coral structure despite changes in tank environment in successive rounds. Given that breeders are large and hence competitively dominant over smaller subordinates, and assuming that corals of higher complexity are considered higher quality, then it is not surprising that breeders would stay in or move to the high complexity after subordinate addition. However, breeders continuing to occupy or choosing to occupy the high complexity coral structure despite the reduction in coral size was not expected given the strong influence of habitat size on habitat choice, fitness, sociality, and group size (Wong et al. 2005; Thompson et al. 2007; Pereira and Munday 2016; Hing et al. 2019; Rueger et al. 2021). The priority of high complexity despite a size reduction suggests that breeders traded off costs due to the reduction in coral size, with benefits accrued from occupying the high complexity coral structure even though the high complexity structure was smaller than the low complexity coral structure. What these benefits of high complexity may be are yet to be confirmed, but may include survival and reproductive advantage gained from reduced predation, increased food availability, and increased fecundity (Wong and Buston 2013; González‐Rivero et al. 2017). However, this was not seen in the subordinates, which appeared to maintain a consistent pattern of occupation across the high complexity, low complexity, and no‐choice categories post the initial exploratory period exhibited.

In this study, the greatest number of switches performed by an individual was three within a 24‐h period. This was more than initially expected due to *Gobiodon* typically being viewed as having high site fidelity and displaying little movement on the reef (Munday 2000; Wall and Herler 2009; Pereira and Munday 2016). Breeders (particularly females) switched more during trial round 1 compared to the latter rounds 2 and 3, where they switched far less. This suggests that breeders may benefit from reduced movement when faced with unstable social dynamics (addition of a subordinate, round 2) and changing environments (reduction in coral size, round 3). These benefits may arise from reduced energy expenditure from movement and avoiding high predation risk during movement (Munday et al. 1998; Froehlich et al. 2022) during times of instability. Conversely, the greater instances of movement during the initial 12 h (round 1) could be beneficial as breeders explored the environment with little perceived predation pressure. After this initial period, breeders then appeared to settle in the higher complexity habitat during the various disturbances, electing to not move. *Gobiodon* have been shown to remain in their home coral despite bleaching, and will travel up to 10 m to return to their chosen coral if translocated (Froehlich et al. 2022). The current study supports these results, as after their initial exploration, breeders appeared to settle in a coral and chose to remain there.

Subordinate individuals, on the other hand, were observed to switch more frequently than breeders and did not display variation in switch rate with changing trial round. These higher rates of movement may suggest an ontogenetic shift in movement patterns in this species. Ontogenetic changes in behavioral traits and patterns from juvenile/recruit ages individuals to adults are not uncommon in *Gobiodon* and other coral dwelling fishes (Kawasaki et al. 2003; Wall and Herler 2009; Wehrberger and Herler 2014; Pereira et al. 2015). A similar movement pattern between subordinates and adult breeders was observed in *Gobiodon histrio* , with subordinates moving between corals at higher rates than mature breeders (Wall and Herler 2009). Wall and Herler (2009) suggested that the increased movement seen in younger non‐paired individuals could be attributed to habitat and/or mate search, which may also explain why the breeder pairs, which were already in a reproductive position, did not switch frequently following their initial choice. Alternatively, or in addition, subordinates may have switched more often because of eviction by the breeder males and females. Eviction of subordinates by the dominant breeding pair is a behavior frequently observed in coral dwelling reef fish (Wong et al. 2007; Wong 2010; Rueger et al. 2021). Therefore, it is not unreasonable that the breeder pair may have evicted the subordinate from the high complexity habitat, in turn increasing the number of switches recorded for the subordinate individuals, especially if there were repeat attempts to enter the high complexity.

Environmental pressures, as a consequence of climate change and increased disturbance regimes of cyclones and bleaching events, may threaten a number of coral reef mutualistic relationships. The relationship between coral gobies (*Gobiodon*) and their coral hosts is one of these mutualisms under threat (Froehlich et al. 2023). *Gobiodon* have exhibited optimal fitness when occupying their most preferred coral species (Pereira and Munday 2016); however, when faced with multiple disturbances with little recovery time, both their social structures and coral occupation have been observed to change (Hing et al. 2018; Froehlich et al. 2023). It has been observed that shortly after the disturbance, the group size and the size of the coral groups they were inhabiting had decreased from pre‐disturbance levels (Hing et al. 2018). Even after a recovery time of 3 years, gobies were observed still inhabiting corals in the post‐disturbance smaller social structures and coral species, often not their preferred host species, rather than returning to their predisturbance occupancy patterns (Froehlich et al. 2023). The shift to non‐optimal coral hosts could have long‐term effects on *Gobiodon* due to the reduction in fitness, hence placing both *Gobiodon* and their mutualistic relationship with host corals in danger as the frequency and severity of disturbances increases. Therefore, understanding the role of coral complexity in the habitat choice and social decisions in this genus is vital in the face of an uncertain future.

While *G. quinquestrigatus* is an ideal study species due to its widespread distribution and habitat generalist nature (Munday et al. 1999), it occurs towards the lower end of the sociality index in most locations (Hing et al. 2019; Froehlich et al. 2024). Other species, such as *G. fuscoruber* and *G. rivulatus* , show social indices that are higher than *G. quinquestrigatus*, suggesting they are more likely to be social (Hing et al. 2019; Froehlich et al. 2024). In the future, conducting similar experiments using these more social species, as well as on less social species such as *G. axillaris* or *G. brochus* , would be highly informative to further investigate the role of complexity and social status on group living choices. Future analyses could also investigate how attachment to the originally chosen host coral in the aquaria influences occupancy decisions in trials such as those conducted in this study. Additionally, future studies could investigate whether it is the structural complexity per se versus the possible indirect benefits associated with coral structural complexity that form the basis of habitat choice decisions. For example, a manipulative experiment comparing clutch size where the breeder female was provided with supplemental food or not while occupying a high or low complexity coral would clarify the role of direct versus indirect benefits of high complexity corals. Furthermore, examining the habitat choices and movement patterns of subordinates in a tank with a high and low complexity coral option with and without a breeder pair present would enable us to investigate the influences of breeder behavior on subordinate choices. Future directions such as these outlined are fundamental in understanding the habitat choice decisions made by obligate mutualists such as coral dwelling fish, and how symbiotic relationships may be altered by environmental pressures such as habitat degradation.

Based on our understanding of how environmental stressors impact coral reefs, combined with the insights into habitat complexity and social dynamics observed in this study, we can consider potential future scenarios for these ecosystems. Coral‐dwelling fish, such as those in the genus *Gobiodon*, are crucial for healthy coral reefs because of their close relationship with scleractinian corals and their capacity to lessen environmental stresses. These fish provide several benefits to corals, including protection from predators (Gochfeld 2010), increased nutrient availability (Chase et al. 2014; Meyer and Schultz 1985a; Shantz and Burkepile 2014), improved water flow and oxygen levels in coral tissues (Berenshtein et al. 2015; Garcia‐Herrera et al. 2017; Goldshmid and Holzman 2004), slower development of coral diseases (Chong‐Seng et al. 2011), and enhanced overall coral growth (Liberman et al. 1995; Meyer and Schultz 1985b; Shantz et al. 2016). Importantly, recent findings indicate that the presence of coral‐dwelling fish significantly improves the survival and recovery of corals undergoing bleaching events caused by higher water temperatures (Chase et al. 2018). Therefore, identifying the specific coral traits that attract these fish could be very valuable for creating effective strategies to mitigate climate change impacts on reefs. By considering the preferences of coral‐dwelling fish in selective breeding programs focused on developing heat‐tolerant corals, we could encourage fish habitation. This, in turn, could increase the long‐term success of coral restoration efforts by providing the additional protective and supportive benefits offered by these mutualistic fish.

Overall, our study highlights the importance of coral complexity in the grouping decisions of coral dwelling gobies. Individuals will predominantly choose a high complexity coral if given the choice. Breeder individuals will continue to inhabit a high complexity coral even if coral size is reduced, trading off coral size for higher complexity. Breeders and subordinate individuals behave differently when choosing and moving between corals of differing complexity, displaying the critical role of social dynamics in the host choices observed. With the mutualisms such as these between gobies (*Gobiodon*) and corals being under threat from environmental pressures leading to decreased habitat quality, it is crucial to understand the habitat choice decisions and sociality patterns in response to coral complexity. Therefore, our study confirms the importance of structural complexity alongside other habitat characteristics that influence the habitat and grouping choices of individuals.

## Author Contributions

**Courtney A. Hildebrandt:** conceptualization (lead), data curation (lead), formal analysis (lead), funding acquisition (equal), investigation (lead), methodology (lead), project administration (equal), validation (equal), visualization (lead), writing – original draft (lead), writing – review and editing (equal). **Catheline Y. M. Froehlich:** funding acquisition (equal), investigation (equal), methodology (equal), writing – review and editing (equal). **O. Selma Klanten:** conceptualization (equal), investigation (equal), methodology (equal), writing – review and editing (equal). **Marian Y. L. Wong:** conceptualization (equal), funding acquisition (lead), investigation (equal), methodology (equal), project administration (lead), supervision (lead), writing – review and editing (equal).

## Ethics Statement

The study follows the relevant guidelines and regulations, including the PREPARE and ARRIVE guidelines. The project was completed under the animal ethics protocols of the University of Wollongong Animal Ethics Committee, protocol project number AE21/18. The collections were conducted in accordance with Great Barrier Reef Marine Park Authority (GBRMPA) Scientific Collection permit number G18/41020.1.

## Conflicts of Interest

The authors declare no conflicts of interest.

## Supporting information


**Data S1:** ece371887‐sup‐0001‐DataS1.zip.

## Data Availability

All data is accessible on the data repository Dryad under the same name as the paper (https://doi.org/10.5061/dryad.dv41ns28f) and has been uploaded as Supporting Information.

## References

[ece371887-bib-0001] Acosta, C. , and D. Robertson . 2002. “Diversity in Coral Reef Fish Communities: The Effects of Habitat Patchiness Revisited.” Marine Ecology Progress Series 227: 87–96. 10.3354/meps227087.

[ece371887-bib-0002] Atwood, T. C. 2006. “The Influence of Habitat Patch Attribute on Coyote Group Size and Interaction in a Fragmented Landscape.” Canadian Journal of Zoology 84: 80–87.

[ece371887-bib-0003] Bateman, A. J. 1948. “Intra‐Sexual Selection in Drosophila.” Heredity 2, no. 3: 349–368. 10.1038/hdy.1948.21. 18103134

[ece371887-bib-0004] Bates, D. , M. Mächler , B. Bolker , and S. Walker . 2015. “Fitting Linear Mixed‐Effects Models Using lme4.” Journal of Statistical Software 67, no. 1: 1–127. 10.18637/jss.v067.i01.

[ece371887-bib-0005] Beese, C. M. , P. J. Mumby , and A. Rogers . 2023. “Small‐Scale Habitat Complexity Preserves Ecosystem Services on Coral Reefs.” Journal of Applied Ecology 60, no. 9: 1854–1867. 10.1111/1365-2664.14458.

[ece371887-bib-0006] Berenshtein, I. , Y. Reuben , and A. Genin . 2015. “Effect of Oxygen on Coral Fanning by Mutualistic Fish.” Marine Ecology 36, no. 4: 1171–1175.

[ece371887-bib-0007] Bonabeau, E. , L. Dagorn , and P. Préon . 1999. “Scaling in Animal Group‐Size Distributions.” Proceedings of the National Academy of Sciences of the United States of America 96, no. 8: 4472–4477. 10.1073/pnas.96.8.4472. 10200286 PMC16356

[ece371887-bib-0008] Brooker, R. M. , P. L. Munday , and T. D. Ainsworth . 2010. “Diets of Coral‐Dwelling Fishes of the Genus Gobiodon With Evidence of Corallivory.” Journal of Fish Biology 76, no. 10: 2578–2583. 10.1111/j.1095-8649.2010.02644.x. 20557610

[ece371887-bib-0009] Buston, P. M. 2003. “Mortality Is Associated With Social Rank in the Clown Anemonefish ( *Amphiprion percula* ).” Marine Biology 143, no. 4: 811–815. 10.1007/s00227-003-1106-8.

[ece371887-bib-0010] Chan, Y. , S. Lo , A. Quan , and D. T. Blumstein . 2019. “Ontogenetic Shifts in Perceptions of Safety Along Structural Complexity Gradients in a Territorial Damselfish.” Current Zoology 65, no. 2: 183–188. 10.1093/cz/zoy091. 30936907 PMC6430967

[ece371887-bib-0011] Chapin, K. J. 2014. “Microhabitat and Spatial Complexity Predict Group Size of the Whip Spider *Heterophrynus Batesii* in Amazonian Ecuador.” Journal of Tropical Ecology 30, no. 2: 173–177. 10.1017/S0266467413000850.

[ece371887-bib-0012] Chase, T. J. , M. S. Pratchett , G. E. Frank , and M. O. Hoogenboom . 2018. “Coral‐Dwelling Fish Moderate Bleaching Susceptibility of Coral Hosts.” PLoS One 13, no. 12: e0208545. 30550591 10.1371/journal.pone.0208545 PMC6294555

[ece371887-bib-0013] Chase, T. J. , M. S. Pratchett , S. P. W. Walker , and M. O. Hoogenboom . 2014. “Small‐Scale Environmental Variation Influences Whether Coral‐Dwelling Fish Promote or Impede Coral Growth.” Oecologia 176, no. 4: 1009–1022. 25205029 10.1007/s00442-014-3065-9

[ece371887-bib-0014] Chong‐Seng, K. M. , A. J. Cole , M. S. Pratchett , and B. L. Willis . 2011. “Selective Feeding by Coral Reef Fishes on Coral Lesions Associated With Brown Band and Black Band Disease.” Coral Reefs 30, no. 2: 473–481.

[ece371887-bib-0015] Clutton‐Brock, T. H. , S. J. Hodge , and T. P. Flower . 2008. “Group Size and the Suppression of Subordinate Reproduction in Kalahari Meerkats.” Animal Behaviour 76, no. 3: 689–700. 10.1016/j.anbehav.2008.03.015.

[ece371887-bib-0016] Coker, D. J. , N. A. J. Graham , and M. S. Pratchett . 2012. “Interactive Effects of Live Coral and Structural Complexity on the Recruitment of Reef Fishes.” Coral Reefs 31, no. 4: 919–927. 10.1007/s00338-012-0920-1.

[ece371887-bib-0017] Darling, E. S. , N. A. J. Graham , F. A. Januchowski‐Hartley , K. L. Nash , M. S. Pratchett , and S. K. Wilson . 2017. “Relationships Between Structural Complexity, Coral Traits, and Reef Fish Assemblages.” Coral Reefs 36, no. 2: 561–575. 10.1007/s00338-017-1539-z.

[ece371887-bib-0018] Dirnwöber, M. , and J. Herler . 2007. “Microhabitat Specialisation and Ecological Consequences for Coral Gobies of the Genus Gobiodon in the Gulf of Aqaba, Northern Red Sea.” Marine Ecology Progress Series 342, no. July: 265–275. 10.3354/meps342265.

[ece371887-bib-0019] Elff, M. 2022. ‘Multinomial Logit Models, with or without Random Effects or Overdispersion’, *CRAN* [Preprint].

[ece371887-bib-0020] Elff, M. 2023. “Management of Survey Data and Presentation of Analysis Results.”

[ece371887-bib-0021] Emslie, M. J. , A. J. Cheal , and K. A. Johns . 2014. “Retention of Habitat Complexity Minimizes Disassembly of Reef Fish Communities Following Disturbance: A Large‐Scale Natural Experiment.” PLoS One 9, no. 8: e105384. 10.1371/journal.pone.0105384. 25140801 PMC4139330

[ece371887-bib-0022] Feary, D. , G. R. Almany , G. P. Jones , and M. McCormick . 2007. “Coral Degradation and the Structure of Tropical Reef Fish Communities.” Marine Ecology Progress Series 333: 243–248. 10.3354/meps333243.

[ece371887-bib-0023] Fox, J. , and S. Weisberg . 2019. An {R} Companion to Applied Regression. Third. Sage.

[ece371887-bib-0024] Froehlich, C. Y. M. , S. J. Heatwole , O. S. Klanten , and M. Y. L. Wong . 2022. “Habitat Health, Size and Saturation Do Not Alter Movement Decisions in a Social Coral Reef Fish.” Animal Behaviour 191: 125–133. 10.1016/j.anbehav.2022.06.015.

[ece371887-bib-0025] Froehlich, C. Y. M. , S. J. Heatwole , O. Selma Klanten , et al. 2024. “Multi‐Level Framework to Assess Social Variation in Response to Ecological and Social Factors: Modeled With Coral Gobies.” Oikos 2024: e10669. 10.1111/oik.10669.

[ece371887-bib-0026] Froehlich, C. Y. M. , O. S. Klanten , M. L. Hing , M. Dowton , and M. Y. L. Wong . 2021. “Uneven Declines Between Corals and Cryptobenthic Fish Symbionts From Multiple Disturbances.” Scientific Reports 11, no. 1: 16420. 10.1038/s41598-021-95778-x. 34385506 PMC8361158

[ece371887-bib-0027] Froehlich, C. Y. M. , O. S. Klanten , M. L. Hing , M. Dowton , and M. Y. L. Wong . 2023. “Delayed Recovery and Host Specialization May Spell Disaster for Coral‐Fish Mutualism.” Ecology and Evolution 13, no. 6: e10209. 10.1002/ece3.10209. 37361899 PMC10285627

[ece371887-bib-0028] Furness, R. W. , and T. R. Birkhead . 1984. “Seabird Colony Distributions Suggest Competition for Food Supplies During the Breeding Season.” Nature 311, no. 5987: 655–656. 10.1038/311655a0.

[ece371887-bib-0029] Garcia‐Herrera, N. , S. C. A. Ferse , A. Kunzmann , and A. Genin . 2017. “Mutualistic Damselfish Induce Higher Photosynthetic Rates in Their Host Coral.” Journal of Experimental Biology 220, no. 10: 1803–1811. 28515171 10.1242/jeb.152462

[ece371887-bib-0030] Gerard, J. F. , and P. Loisel . 1995. “Spontaneous Emergence of a Relationship Between Habitat Openness and Mean Group Size and Its Possible Evolutionary Consequences in Large Herbivores.” Journal of Theoretical Biology 176, no. 4: 511–522. 10.1006/jtbi.1995.0217.

[ece371887-bib-0031] Gillespie, T. , and C. Chapman . 2001. “Determinants of Group Size in the Red Colobus Monkey ( *Procolobus badius* ): An Evaluation of the Generality of the Ecological‐Constraints Model.” Behavioral Ecology and Sociobiology 50, no. 4: 329–338. 10.1007/s002650100371.

[ece371887-bib-0032] Gochfeld, D. 2010. “Territorial Damselfishes Facilitate Survival of Corals by Providing an Associational Defense Against Predators.” Marine Ecology Progress Series 398: 137–148.

[ece371887-bib-0033] Goiran, C. , and R. Shine . 2015. “Parental Defence on the Reef: Antipredator Tactics of Coral‐Reef Fishes Against Egg‐Eating Seasnakes: Parental Defence on the Reef.” Biological Journal of the Linnean Society 114, no. 2: 415–425. 10.1111/bij.12422.

[ece371887-bib-0034] Goldshmid, R. , and R. Holzman . 2004. “Aeration of Corals by Sleep‐Swimming Fish.” Limnology and Oceanography 49, no. 5: 1832–1839.

[ece371887-bib-0035] González‐Rivero, M. , A. R. Harborne , A. Herrera‐Reveles , et al. 2017. “Linking Fishes to Multiple Metrics of Coral Reef Structural Complexity Using Three‐Dimensional Technology.” Scientific Reports 7, no. 1: 13965. 10.1038/s41598-017-14272-5. 29070893 PMC5656654

[ece371887-bib-0036] Gratwicke, B. , and M. R. Speight . 2005. “The Relationship Between Fish Species Richness, Abundance and Habitat Complexity in a Range of Shallow Tropical Marine Habitats.” Journal of Fish Biology 66: 650–667. 10.1111/j.0022-1112.2005.00629.x.

[ece371887-bib-0037] Hernaman, V. , and P. L. Munday . 2007. “Evolution of Mating Systems in Coral Reef Gobies and Constraints on Mating System Plasticity.” Coral Reefs 26, no. 3: 585–595. 10.1007/s00338-007-0222-1.

[ece371887-bib-0038] Hing, M. L. , O. S. Klanten , M. Dowton , K. R. Brown , and M. Y. L. Wong . 2018. “Repeated Cyclone Events Reveal Potential Causes of Sociality in Coral‐Dwelling Gobiodon Fishes.” PLoS One 13, no. 9: e0202407. 10.1371/journal.pone.0202407. 30183723 PMC6124712

[ece371887-bib-0039] Hing, M. L. , O. S. Klanten , M. Y. L. Wong , and M. Dowton . 2019. “Drivers of Sociality in Gobiodon Fishes: An Assessment of Phylogeny, Ecology and Life‐History.” Molecular Phylogenetics and Evolution 137, no. May: 263–273. 10.1016/j.ympev.2019.05.020. 31125658

[ece371887-bib-0040] Holbrook, S. J. , A. J. Brooks , and R. J. Schmitt . 2002. “Predictability of Fish Assemblages on Coral Patch Reefs.” Marine and Freshwater Research 53, no. 2: 181. 10.1071/MF01137.

[ece371887-bib-0041] Holbrook, S. J. , G. E. Forrester , and R. J. Schmitt . 2000. “Spatial Patterns in Abundance of a Damselfish Reflect Availability of Suitable Habitat.” Oecologia 122, no. 1: 109–120. 10.1007/PL00008826. 28307947

[ece371887-bib-0042] Janson, C. H. , and M. L. Goldsmith . 1995. “Predicting Group Size in Primates: Foraging Costs and Predation Risks.” Behavioral Ecology 6, no. 3: 326–336. 10.1093/beheco/6.3.326.

[ece371887-bib-0043] Jordan, L. K. B. , D. S. Gilliam , and R. E. Spieler . 2005. “Reef Fish Assemblage Structure Affected by Small‐Scale Spacing and Size Variations of Artificial Patch Reefs.” Journal of Experimental Marine Biology and Ecology 326, no. 2: 170–186. 10.1016/j.jembe.2005.05.023.

[ece371887-bib-0044] Kawasaki, H. , M. Sano , and T. Shibuno . 2003. “The Relationship Between Habitat Physical Complexity and Recruitment of the Coral Reef Damselfish, *Pomacentrus amboinensis* : An Experimental Study Using Small‐Scale Artificial Reefs.” Ichthyological Research 50: 73–77.

[ece371887-bib-0045] Kokko, H. , and M. Jennions . 2003. “It Takes Two to Tango.” Trends in Ecology & Evolution 18, no. 3: 103–105.

[ece371887-bib-0046] Kokko, H. , and M. D. Jennions . 2008. “Parental Investment, Sexual Selection and Sex Ratios.” Journal of Evolutionary Biology 21, no. 4: 919–948. 10.1111/j.1420-9101.2008.01540.x. 18462318

[ece371887-bib-0047] Komyakova, V. , P. L. Munday , and G. P. Jones . 2013. “Relative Importance of Coral Cover, Habitat Complexity and Diversity in Determining the Structure of Reef Fish Communities.” PLoS One 8, no. 12: 1–12. 10.1371/journal.pone.0083178. PMC3862682 24349455

[ece371887-bib-0048] Kuznetsova, A. , P. B. Brockhoff , and R. H. Christensen . 2017. “lmerTest Package: Tests in Linear Mixed Effects Models.” Journal of Statistical Software 82, no. 13: 1–31. 10.18637/jss.v082.i13.

[ece371887-bib-0049] Lawrence, A. , K. O'Connor , V. Haroutounian , and A. Swei . 2018. “Patterns of Diversity Along a Habitat Size Gradient in a Biodiversity Hotspot.” Ecosphere 9, no. 4: e02183. 10.1002/ecs2.2183.

[ece371887-bib-0050] Liberman, T. , A. Genin , and Y. Loya . 1995. “Effects on Growth and Reproduction of the Coral *Stylophora pistillata* by the Mutualistic Damselfish *Dascyllus marginatus* .” Marine Biology 121, no. 4: 741–746.

[ece371887-bib-0051] Lüdecke, D. 2023. “sjPlot: Data Visualization for Statistics in Social Science.” https://CRAN.R‐project.org/package=sjPlot.

[ece371887-bib-0052] Meyer, J. L. , and E. T. Schultz . 1985a. “Migrating Haemulid Fishes as a Source of Nutrients and Organic Matter on Coral Reefs: N, P, and POC From Reef Fish.” Limnology and Oceanography 30, no. 1: 146–156.

[ece371887-bib-0053] Meyer, J. L. , and E. T. Schultz . 1985b. “Tissue Condition and Growth Rate of Corals Associated With Schooling Fish: Effects of Fish on Corals.” Limnology and Oceanography 30, no. 1: 157–166.

[ece371887-bib-0054] Mills, M. S. , T. Schils , A. D. Olds , and J. X. Leon . 2023. “Structural Complexity of Coral Reefs in Guam, Mariana Islands.” Remote Sensing 15: 5558. 10.3390/rs15235558.

[ece371887-bib-0055] Munday, P. L. 2000. “Interactions Between Habitat Use and Patterns of Abundance in Coral‐Dwelling Fishes of the Genus Gobiodon.” Environmental Biology of Fishes 58, no. 4: 355–369. 10.1023/A:1007689314926.

[ece371887-bib-0056] Munday, P. L. , M. J. Caley , and G. P. Jones . 1998. “Bi‐Directional Sex Change in a Coral‐Dwelling Goby.” Behavioral Ecology and Sociobiology 43: 371–377.

[ece371887-bib-0057] Munday, P. L. , A. S. Harold , and R. Winterbottom . 1999. “Guide to Coral‐Dwelling Gobies, Genus Gobiodon (Gobiidae) From Papua New Guinea and the Great Barrier Reef.” Revue Française D'aquariologie Herpétologie 26, no. 1–2: 53–58.

[ece371887-bib-0058] Munday, P. L. , and S. K. Wilson . 1997. “Comparative Efficacy of Clove Oil and Other Chemicals in Anaesthetization of *Pomacentrus amboinensis*, a Coral Reef Fish.” Journal of Fish Biology 51, no. 5: 931–938. 10.1006/jfbi.1997.0498.

[ece371887-bib-0059] Noonan, S. H. C. , G. P. Jones , and M. S. Pratchett . 2012. “Coral Size, Health and Structural Complexity: Effects on the Ecology of a Coral Reef Damselfish.” Marine Ecology Progress Series 456: 127–137. 10.3354/meps09687.

[ece371887-bib-0060] Parker, G. A. 1970. “Sperm Competition and Its Evolutionary Consequences in Insects.” Biological Reviews 45, no. 4: 525–567. 10.1111/j.1469-185X.1970.tb01176.x.

[ece371887-bib-0061] Parker, G. A. 1982. “Why Are There So Many Tiny Sperm? Sperm Competition and the Maintenance of Two Sexes.” Journal of Theoretical Biology 96, no. 2: 281–294. 7121030 10.1016/0022-5193(82)90225-9

[ece371887-bib-0062] Pereira, P. H. C. , and P. L. Munday . 2016. “Coral Colony Size and Structure as Determinants of Habitat Use and Fitness of Coral‐Dwelling Fishes.” Marine Ecology Progress Series 553, no. July: 163–172. 10.3354/meps11745.

[ece371887-bib-0063] Pereira, P. H. C. , P. L. Munday , and G. P. Jones . 2015. “Competitive Mechanisms Change With Ontogeny in Coral‐Dwelling Gobies.” Ecology 96, no. 11: 3090–3101. 27070026 10.1890/14-1689.1

[ece371887-bib-0064] R Core Team . 2021. “R: A language and environment for statistical computing. Vienna, Austria: R Foundation for Statistical Computing.” https://www.R‐project.org/.

[ece371887-bib-0065] Reed, B. , and K. Hovel . 2006. “Seagrass Habitat Disturbance: How Loss and Fragmentation of Eelgrass *Zostera marina* Influences Epifaunal Abundance and Diversity.” Marine Ecology Progress Series 326: 133–143. 10.3354/meps326133.

[ece371887-bib-0066] Ricklefs, R. E. , and I. J. Lovette . 1999. “The Roles of Island Area *Per Se* and Habitat Diversity in the Species–Area Relationships of Four Lesser Antillean Faunal Groups.” Journal of Animal Ecology 68, no. 6: 1142–1160. 10.1046/j.1365-2656.1999.00358.x.

[ece371887-bib-0067] Rueger, T. , R. Branconi , C. Y. Froehlich , S. J. Heatwole , M. Y. Wong , and P. M. Buston . 2021. “The Next Frontier in Understanding the Evolution of Coral Reef Fish Societies.” Frontiers in Marine Science 8: 665780. 10.3389/fmars.2021.665780.

[ece371887-bib-0068] Sale, P. F. , W. A. Douglas , and P. J. Doherty . 1984. “Choice of Microhabitats by Coral Reef Fishes at Settlement.” Coral Reefs 3, no. 2: 91–99. 10.1007/BF00263759.

[ece371887-bib-0069] Schiemer, L. , S. Niedermüller , and J. Herler . 2009. “The Influence of Colony Size and Coral Health on the Occupation of Coral‐Associated Gobies (Pisces: Gobiidae).” Coral Reefs 28, no. 1: 137–142. 10.1007/s00338-008-0420-5.

[ece371887-bib-0070] Schneider, C. A. , W. S. Rasband , and K. W. Eliceiri . 2012. “NIH Image to ImageJ: 25 Years of Image Analysis.” Nature Methods 9, no. 7: 671–675. 22930834 10.1038/nmeth.2089 PMC5554542

[ece371887-bib-0071] Schroeder, R. E. 1987. “Effects of Patch Reef Size and Isolation on Coral Reef Fish Recruitment.” Bulletin of Marine Science 41, no. 2: 441–451.

[ece371887-bib-0072] Schuler, M. S. , J. M. Chase , and T. M. Knight . 2017. “Habitat Size Modulates the Influence of Heterogeneity on Species Richness Patterns in a Model Zooplankton Community.” Ecology 98, no. 6: 1651–1659. 10.1002/ecy.1833. 28369846

[ece371887-bib-0073] Shantz, A. A. , and D. E. Burkepile . 2014. “Context‐Dependent Effects of Nutrient Loading on the Coral–Algal Mutualism.” Ecology 95, no. 7: 1995–2005. 25163130 10.1890/13-1407.1

[ece371887-bib-0074] Shantz, A. A. , M. C. Ladd , E. Schrack , and D. E. Burkepile . 2016. “Fish‐Derived Nutrient Hotspots Shape Coral Reef Benthic Communities.” Ecological Applications 25, no. 8: 2142–2152. 10.1890/14-2209.1 26910945

[ece371887-bib-0075] Thompson, V. J. , P. L. Munday , and G. P. Jones . 2007. “Habitat Patch Size and Mating System as Determinants of Social Group Size in Coral‐Dwelling Fishes.” Coral Reefs 26, no. 1: 165–174. 10.1007/s00338-006-0181-y.

[ece371887-bib-0076] Untersteggaber, L. , P. Mitteroecker , and J. Herler . 2014. “Coral Architecture Affects the Habitat Choice and Form of Associated Gobiid Fishes.” Marine Biology 161, no. 3: 521–530. 10.1007/s00227-013-2354-x. 24587541 PMC3931935

[ece371887-bib-0077] Venables, W. N. , B. D. Ripley , and W. N. Venables . 2002. Modern Applied Statistics With S. 4th ed. Springer (Statistics and computing).

[ece371887-bib-0078] Wall, M. , and J. Herler . 2009. “Postsettlement Movement Patterns and Homing in a Coral‐Associated Fish.” Behavioral Ecology 20, no. 1: 87–95. 10.1093/beheco/arn118.

[ece371887-bib-0079] Wehrberger, F. , and J. Herler . 2014. “Microhabitat Characteristics Influence Shape and Size of Coral‐Associated Fishes.” Marine Ecology Progress Series 500: 203–214. 10.3354/meps10689.

[ece371887-bib-0080] Whiteman, E. A. , and I. M. Côté . 2004. “Monogamy in Marine Fishes.” Biological Reviews 79, no. 2: 351–375. 10.1017/S1464793103006304. 15191228

[ece371887-bib-0081] Wong, M. Y. L. 2010. “Ecological Constraints and Benefits of Philopatry Promote Group‐Living in a Social but Non‐Cooperatively Breeding Fish.” Proceedings of the Royal Society B: Biological Sciences 277, no. 1680: 353–358. 10.1098/rspb.2009.1453. PMC2842644 19828547

[ece371887-bib-0082] Wong, M. Y. L. , P. M. Buston , P. L. Munday , and G. P. Jones . 2007. “The Threat of Punishment Enforces Peaceful Cooperation and Stabilizes Queues in a Coral‐Reef Fish.” Proceedings of the Royal Society B: Biological Sciences 274, no. 1613: 1093–1099. 10.1098/rspb.2006.0284. PMC2124475 17301018

[ece371887-bib-0083] Wong, M. Y. L. , C. Fauvelot , S. Planes , and P. M. Buston . 2012. “Discrete and Continuous Reproductive Tactics in a Hermaphroditic Society.” Animal Behaviour 84, no. 4: 897–906. 10.1016/j.anbehav.2012.07.013.

[ece371887-bib-0084] Wong, M. Y. L. , P. L. Munday , P. M. Buston , and G. P. Jones . 2008. “Monogamy When There Is Potential for Polygyny: Tests of Multiple Hypotheses in a Group‐Living Fish.” Behavioral Ecology 19, no. 2: 353–361. 10.1093/beheco/arm141.

[ece371887-bib-0085] Wong, M. Y. L. , P. L. Munday , and G. P. Jones . 2005. “Habitat Patch Size, Facultative Monogamy and Sex Change in a Coral‐Dwelling Fish, *Caracanthus unipinna* .” Environmental Biology of Fishes 74, no. 2: 141–150. 10.1007/s10641-005-6715-2.

[ece371887-bib-0086] Wong, M. Y. L. L. , and P. M. Buston . 2013. “Social Systems in Habitat‐Specialist Reef Fishes: Key Concepts in Evolutionary Ecology.” Bioscience 63, no. 6: 453–463. 10.1525/bio.2013.63.6.7.

