# Supplementary material for: Complex Residences and Sociality: How Coral Structure and Social Environment Influence Occupation Patterns in Gobiodon in Aquaria
Source: Ecol Evol. 2025 Jul 28;15(8):e71887. doi: 10.1002/ece3.71887 (PMC12304431; doi:10.1002/ece3.71887)
Supplement: Supplementary file 1 — Data S1: ece371887‐sup‐0001‐DataS1.zip. [file ECE3-15-e71887-s001.zip › ece371887-sup-0005-R_Output.pdf]

Experimental Choice Data

Courtney A Hildebrandt

2025-01-09

```
#Set working directory
setwd("C:/Users/court/OneDrive/Desktop/OTI Binary Choice")

#Call packages
library(lme4)

## Loading required package: Matrix

library(lmerTest)

##
## Attaching package: 'lmerTest'

## The following object is masked from 'package:lme4':
##
##     lmer

## The following object is masked from 'package:stats':
##
##     step

library(car)

## Warning: package 'car' was built under R version 4.4.2

## Loading required package: carData

library(mclogit)
library(nnet)
library(memisc)

## Warning: package 'memisc' was built under R version 4.4.2

## Loading required package: lattice

## Loading required package: MASS

##
## Attaching package: 'memisc'

## The following object is masked from 'package:car':
##
##     recode
```

```
## The following object is masked from 'package:Matrix':
##
##     as.array

## The following objects are masked from 'package:stats':
##
##     contr.sum, contr.treatment, contrasts

## The following object is masked from 'package:base':
##
##     as.array

library(sjPlot)


#CHOICE FOR MALE, FEMALE AND SUBORDINATE ROUND 3 ONWARDS WHEN SUB INCLUDED -
comparing sex
#Read .csv file
choiceMFS = read.csv("choiceMFS.csv")

#Check variable status
summary(choiceMFS)

##      Trial            Age              Sex.Fref           Sex.Mref
##  Min.   : 1.00   Length:192         Length:192         Length:192
##  1st Qu.: 4.75   Class :character   Class :character   Class :character
##  Median : 8.50   Mode  :character   Mode  :character   Mode  :character
##  Mean   : 8.50
##  3rd Qu.:12.25
##  Max.   :16.00
##    Sex.Sref            Round           choice.highref     choice.nochoicer
ef
##  Length:192         Length:192         Length:192         Length:192
##  Class :character   Class :character   Class :character   Class :character
##  Mode  :character   Mode  :character   Mode  :character   Mode  :character
##
##
##
##  choice.lowref
##  Length:192
##  Class :character
##  Mode  :character
##
##
##

#Reassign variable status
choiceMFS$choice.highref = as.factor(choiceMFS$choice.highref)
choiceMFS$choice.nochoiceref = as.factor(choiceMFS$choice.nochoiceref)
choiceMFS$choice.lowref = as.factor(choiceMFS$choice.lowref)
choiceMFS$Round = as.factor(choiceMFS$Round)
```

```
choiceMFS$Age = as.factor(choiceMFS$Age)
choiceMFS$Sex.Fref = as.factor(choiceMFS$Sex.Fref)
choiceMFS$Sex.Mref = as.factor(choiceMFS$Sex.Mref)
choiceMFS$Sex.Sref = as.factor(choiceMFS$Sex.Sref)
choiceMFS$Trial = as.factor(choiceMFS$Trial)

#Recheck variable status
summary(choiceMFS)

##      Trial              Age      Sex.Fref Sex.Mref  Sex.Sref
##  1       : 12   Adult      :128   F:64     1) M:64   1) S:64
##  2       : 12   Subordinate: 64   M:64     2) F:64   2) F:64
##  3       : 12                     S:64     3) S:64   3) M:64
##  4       : 12
##  5       : 12
##  6       : 12
##  (Other):120
##                                    Round      choice.highref
##  3) Subordinate 12HR                  :48   high      :140
##  4) Subordinate 24HR/Coral Change 0HR:48   low       : 33
##  5) Change 12HR                       :48   no choice: 19
##  6) Change 24HR                       :48
##
##
##
##      choice.nochoiceref       choice.lowref
##  0 - no choice: 19      0 - low       : 33
##  1 - low      : 33      1 - no choice: 19
##  2 - high     :140      2 - high      :140
##
##
##
##

# FEMALE REF
#Define the model with High as the reference category
modelMFSrefFemhigh = mblogit(formula = choice.highref ~ Sex.Fref,
                             random = ~1|Trial,
                             data = choiceMFS)

## Warning: Inner iterations did not coverge - nlminb message: false converge
nce
## (8)

##
## Iteration 1 - deviance = 270.1535 - criterion = 0.8381157

## Warning: Inner iterations did not coverge - nlminb message: false converge
nce
## (8)
```

```
##
## Iteration 2 - deviance = 262.9199 - criterion = 0.03525264

## Warning: Inner iterations did not coverge - nlminb message: false converge
nce
## (8)

##
## Iteration 3 - deviance = 260.8225 - criterion = 0.002925918

## Warning: Inner iterations did not coverge - nlminb message: false converge
nce
## (8)

##
## Iteration 4 - deviance = 260.1095 - criterion = 8.071178e-06

## Warning: Inner iterations did not coverge - nlminb message: false converge
nce
## (8)

##
## Iteration 5 - deviance = 260.0693 - criterion = 5.963892e-11
## converged

#Call results
summary(modelMFSrefFemhigh)

##
## Call:
## mblogit(formula = choice.highref ~ Sex.Fref, data = choiceMFS,
##     random = ~1 | Trial)
##
## Equation for low vs high:
##             Estimate Std. Error z value Pr(>|z|)
## (Intercept)  -2.9088     0.6093  -4.774  1.8e-06 ***
## Sex.FrefM     1.3063     0.6402   2.041 0.041297 *
## Sex.FrefS     2.0538     0.6182   3.322 0.000893 ***
##
## Equation for no choice vs high:
##             Estimate Std. Error z value Pr(>|z|)
## (Intercept)  -2.7245     0.5772  -4.720 2.36e-06 ***
## Sex.FrefM     0.6237     0.6444   0.968    0.333
## Sex.FrefS     0.8649     0.6497   1.331    0.183
## ---
## Signif. codes:  0 '***' 0.001 '**' 0.01 '*' 0.05 '.' 0.1 ' ' 1
##
## (Co-)Variances:
## Grouping level: Trial
##             Estimate        Std.Err.
## low~1       1.2291          1.537
## no choice~1 0.8245 1.3179   1.661 1.761
```

```
##
## Approximate residual deviance: 260.1
## Number of Fisher scoring iterations:  5
## Number of observations
##   Groups by Trial: 16
##   Individual observations:  192
```

```
plot_model(modelMFSrefFemhigh)
```

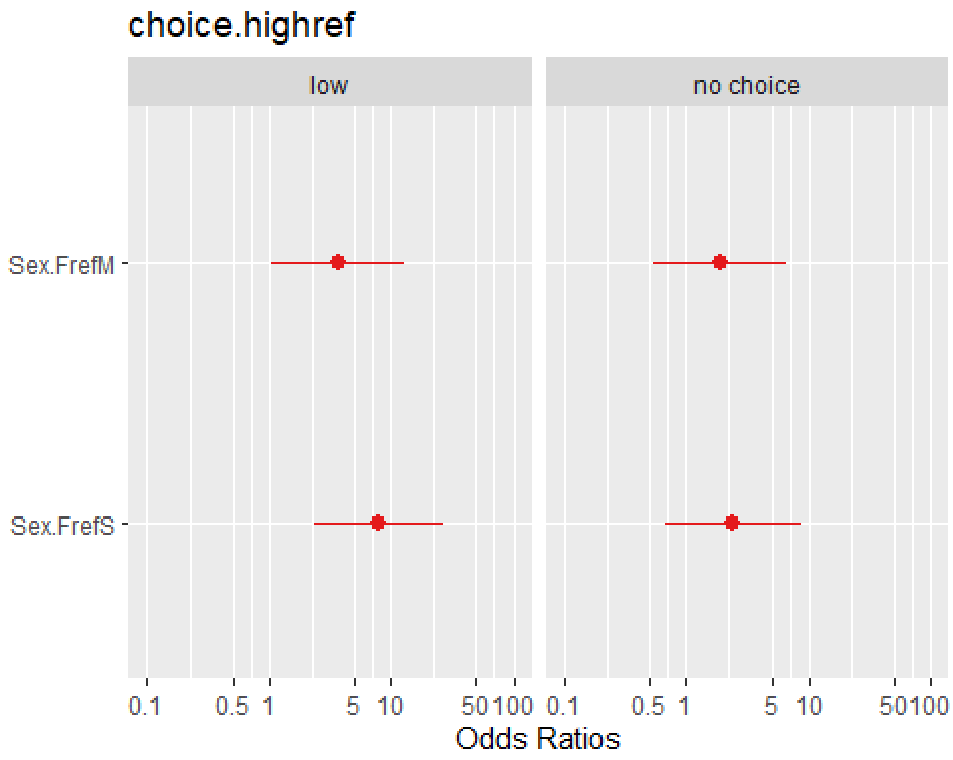


```
tab_model(modelMFSrefFemhigh)
```

	choice.highref: low			choice.highref: no choice		
Predictors	*Odds Ratios*	*CI*	*p*	*Odds Ratios*	*CI*	*p*
(Intercept)	0.05	0.02 – 0.18	**<0.001**	0.07	0.02 – 0.20	**<0.001**
Sex.FrefM	3.69	1.05 – 13.00	**0.041**	1.87	0.53 – 6.62	0.333
Sex.FrefS	7.80	2.31 – 26.29	**0.001**	2.37	0.66 – 8.52	0.183
N_{Trial}	16					
Observations	192					

```r
#Define the model with no choice as the reference category
modelMFSrefFemnochoice = mblogit(formula = choice.nochoiceref ~ Sex.Fref,
                                 random = ~1|Trial,
                                 data = choiceMFS)
```

```
## Warning: Inner iterations did not coverge - nlminb message: false converge
nce
## (8)

##
## Iteration 1 - deviance = 269.953 - criterion = 0.7237408

## Warning: Inner iterations did not coverge - nlminb message: false converge
nce
## (8)

##
## Iteration 2 - deviance = 262.4867 - criterion = 0.04874487

## Warning: Inner iterations did not coverge - nlminb message: false converge
nce
## (8)

##
## Iteration 3 - deviance = 260.675 - criterion = 0.001510352

## Warning: Inner iterations did not coverge - nlminb message: false converge
nce
## (8)

##
## Iteration 4 - deviance = 260.169 - criterion = 2.133962e-06

## Warning: Inner iterations did not coverge - nlminb message: false converge
nce
## (8)

##
## Iteration 5 - deviance = 260.1496 - criterion = 4.680637e-12
## converged
```

```r
#Call results
summary(modelMFSrefFemnochoice)
```

```
##
## Call:
## mblogit(formula = choice.nochoiceref ~ Sex.Fref, data = choiceMFS,
##     random = ~1 | Trial)
##
## Equation for 1 - low vs 0 - no choice:
##             Estimate Std. Error z value Pr(>|z|)
## (Intercept)  -0.2819     0.7058  -0.399    0.690
```

```
## Sex.FrefM     0.6921     0.8311   0.833    0.405
## Sex.FrefS     1.2015     0.8105   1.482    0.138
##
## Equation for 2 - high vs 0 - no choice:
##             Estimate Std. Error z value Pr(>|z|)
## (Intercept)   2.6597     0.5563   4.781 1.74e-06 ***
## Sex.FrefM    -0.6240     0.6400  -0.975    0.330
## Sex.FrefS    -0.8705     0.6453  -1.349    0.177
## ---
## Signif. codes:  0 '***' 0.001 '**' 0.01 '*' 0.05 '.' 0.1 ' ' 1
##
## (Co-)Variances:
## Grouping level: Trial
##            Estimate        Std.Err.
## 1 - low~1  0.4925          0.06368
## 2 - high~1 0.1095 1.0862   0.18676 0.73485
##
## Approximate residual deviance: 260.1
## Number of Fisher scoring iterations:  5
## Number of observations
##   Groups by Trial: 16
##   Individual observations:  192
```

```
plot_model(modelMFSrefFemnochoice)
```

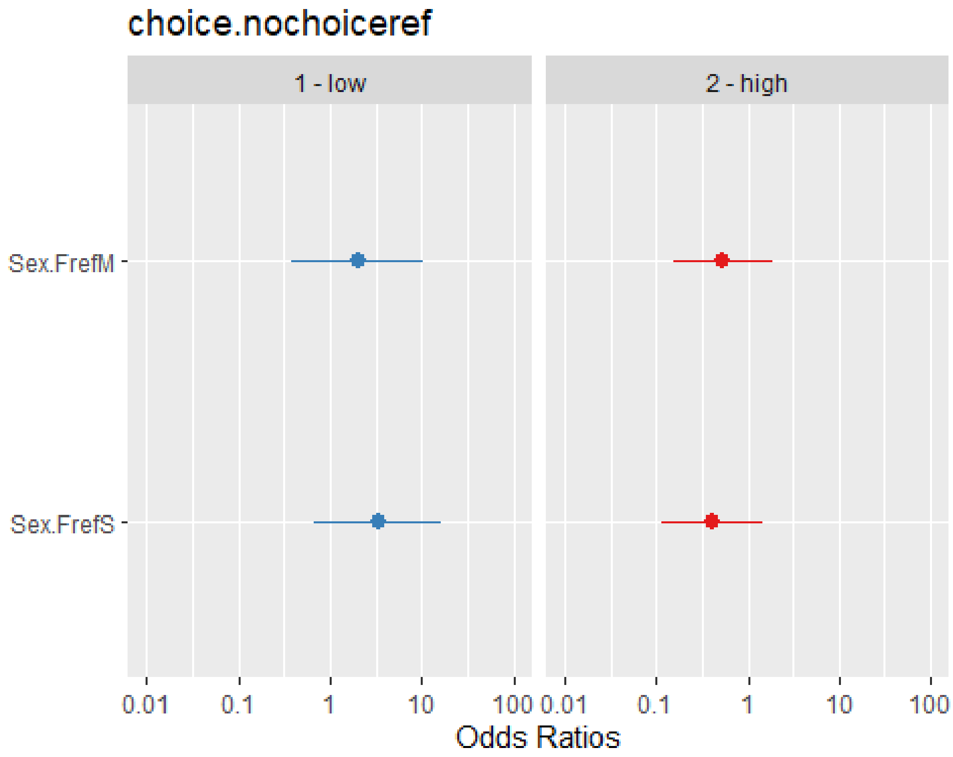


```
tab_model(modelMFSrefFemnochoice)
```

	choice.nochoiceref: 1 - low			choice.nochoiceref: 2 - high		
Predictors	*Odds Ratios*	*CI*	*p*	*Odds Ratios*	*CI*	*p*
(Intercept)	0.75	0.19 – 3.02	0.690	14.29	4.79 – 42.67	**<0.001**
Sex.FrefM	2.00	0.39 – 10.24	0.405	0.54	0.15 – 1.89	0.330
Sex.FrefS	3.33	0.68 – 16.37	0.138	0.42	0.12 – 1.49	0.177
N_{Trial}	16					
Observations	192					

```
# MALE REF
#Define the model with High as the reference category
modelMFSrefMalehigh = mblogit(formula = choice.highref ~ Sex.Mref,
                              random = ~1|Trial,
                              data = choiceMFS)

## Warning: Inner iterations did not coverge - nlminb message: false converge
nce
## (8)

##
## Iteration 1 - deviance = 270.1535 - criterion = 0.8381157

## Warning: Inner iterations did not coverge - nlminb message: false converge
nce
## (8)

##
## Iteration 2 - deviance = 262.9199 - criterion = 0.03525264

## Warning: Inner iterations did not coverge - nlminb message: false converge
nce
## (8)

##
## Iteration 3 - deviance = 260.8225 - criterion = 0.002925918

## Warning: Inner iterations did not coverge - nlminb message: false converge
nce
## (8)

##
## Iteration 4 - deviance = 260.1095 - criterion = 8.071178e-06
```

```
## Warning: Inner iterations did not coverge - nlminb message: false converge
nce
## (8)

##
## Iteration 5 - deviance = 260.0693 - criterion = 5.963892e-11
## converged
```

```
#Call results
summary(modelMFSrefMalehigh)
```

```
##
## Call:
## mblogit(formula = choice.highref ~ Sex.Mref, data = choiceMFS,
##     random = ~1 | Trial)
##
## Equation for low vs high:
##                 Estimate Std. Error z value Pr(>|z|)
## (Intercept)      -1.6025     0.4562  -3.513 0.000444 ***
## Sex.Mref2) F    -1.3063     0.6402  -2.041 0.041297 *
## Sex.Mref3) S     0.7475     0.4698   1.591 0.111620
##
## Equation for no choice vs high:
##                 Estimate Std. Error z value Pr(>|z|)
## (Intercept)      -2.1009     0.5222  -4.023 5.74e-05 ***
## Sex.Mref2) F    -0.6237     0.6444  -0.968    0.333
## Sex.Mref3) S     0.2412     0.6031   0.400    0.689
## ---
## Signif. codes:  0 '***' 0.001 '**' 0.01 '*' 0.05 '.' 0.1 ' ' 1
##
## (Co-)Variances:
## Grouping level: Trial
##              Estimate        Std.Err.
## low~1        1.2291          1.537
## no choice~1 0.8245 1.3179   1.661 1.761
##
## Approximate residual deviance: 260.1
## Number of Fisher scoring iterations:  5
## Number of observations
##   Groups by Trial: 16
##   Individual observations:  192
```

```
plot_model(modelMFSrefMalehigh)
```

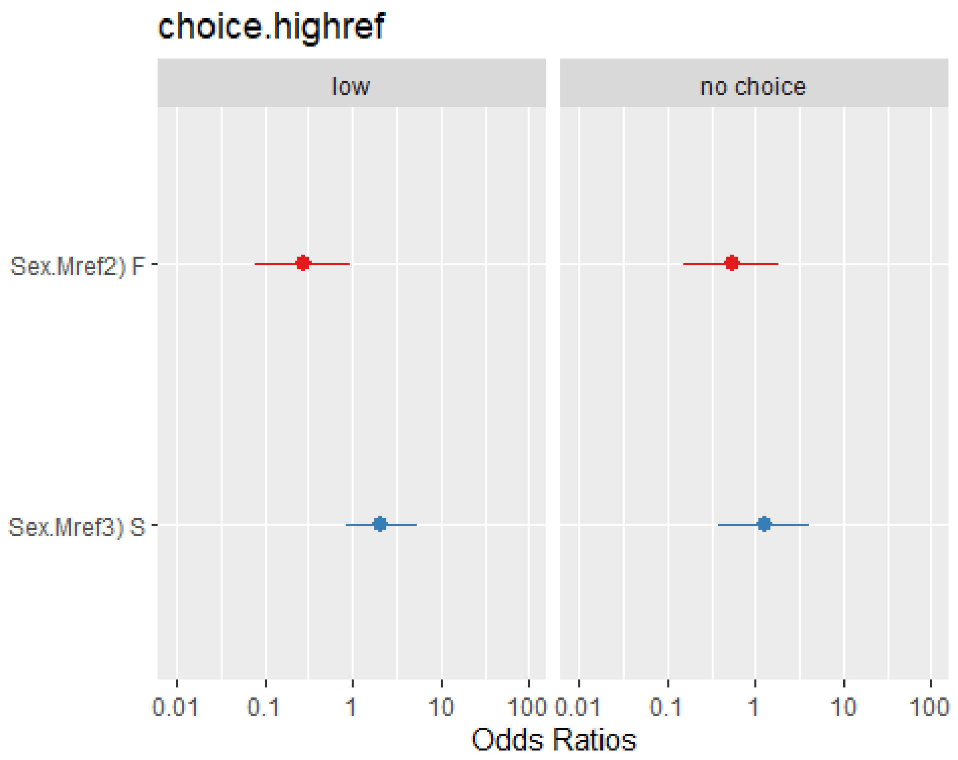


```r
tab_model(modelMFSrefMalehigh)
```

	choice.highref: low			choice.highref: no choice		
Predictors	*Odds Ratios*	*CI*	*p*	*Odds Ratios*	*CI*	*p*
(Intercept)	0.20	0.08 – 0.49	**<0.001**	0.12	0.04 – 0.34	**<0.001**
Sex.Mref2) F	0.27	0.08 – 0.95	**0.041**	0.54	0.15 – 1.90	0.333
Sex.Mref3) S	2.11	0.84 – 5.32	0.112	1.27	0.39 – 4.17	0.689
N_{Trial}	16					
Observations	192					

```r
#Define the model with no choice as the reference category
modelMFSrefMalenochoice = mblogit(formula = choice.nochoiceref ~ Sex.Mref,
                                  random = ~1|Trial,
                                  data = choiceMFS)

## Warning: Inner iterations did not coverge - nlminb message: false converge
nce
## (8)
```

```
##
## Iteration 1 - deviance = 269.953 - criterion = 0.7237408

## Warning: Inner iterations did not coverge - nlminb message: false converge
nce
## (8)

##
## Iteration 2 - deviance = 262.4867 - criterion = 0.04874487

## Warning: Inner iterations did not coverge - nlminb message: false converge
nce
## (8)

##
## Iteration 3 - deviance = 260.675 - criterion = 0.001510352

## Warning: Inner iterations did not coverge - nlminb message: false converge
nce
## (8)

##
## Iteration 4 - deviance = 260.169 - criterion = 2.133962e-06

## Warning: Inner iterations did not coverge - nlminb message: false converge
nce
## (8)

##
## Iteration 5 - deviance = 260.1496 - criterion = 4.680637e-12
## converged

#Call results
summary(modelMFSrefMalenochoice)

##
## Call:
## mblogit(formula = choice.nochoiceref ~ Sex.Mref, data = choiceMFS,
##     random = ~1 | Trial)
##
## Equation for 1 - low vs 0 - no choice:
##                Estimate Std. Error z value Pr(>|z|)
## (Intercept)      0.4102     0.5258   0.780    0.435
## Sex.Mref2) F    -0.6921     0.8311  -0.833    0.405
## Sex.Mref3) S     0.5094     0.6621   0.769    0.442
##
## Equation for 2 - high vs 0 - no choice:
##                Estimate Std. Error z value Pr(>|z|)
## (Intercept)      2.0358     0.5003   4.069 4.71e-05 ***
## Sex.Mref2) F     0.6240     0.6400   0.975     0.33
## Sex.Mref3) S    -0.2465     0.5984  -0.412     0.68
## ---
```

```
## Signif. codes:  0 '***' 0.001 '**' 0.01 '*' 0.05 '.' 0.1 ' ' 1
##
## (Co-)Variances:
## Grouping level: Trial
##              Estimate        Std.Err.
## 1 - low~1  0.4925          0.06368
## 2 - high~1 0.1095 1.0862   0.18676 0.73485
##
## Approximate residual deviance: 260.1
## Number of Fisher scoring iterations:  5
## Number of observations
##   Groups by Trial: 16
##   Individual observations:  192
```

```
plot_model(modelMFSrefMalenochoice)
```

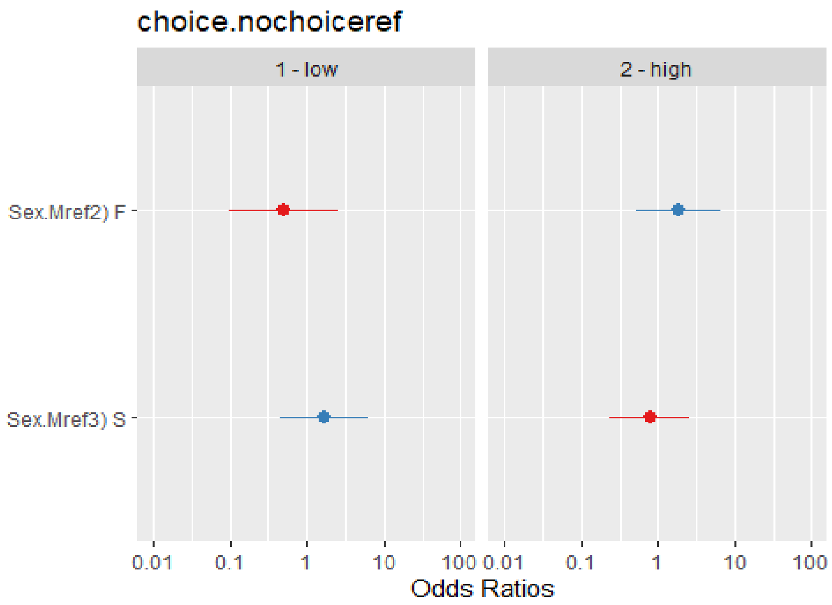


```
tab_model(modelMFSrefMalenochoice)
```

	choice.nochoiceref: 1 - low			choice.nochoiceref: 2 - high		
Predictors	*Odds Ratios*	*CI*	*p*	*Odds Ratios*	*CI*	*p*
(Intercept)	1.51	0.54 – 4.24	0.435	7.66	2.86 – 20.48	**<0.001**
Sex.Mref2) F	0.50	0.10 – 2.57	0.405	1.87	0.53 – 6.57	0.330
Sex.Mref3) S	1.66	0.45 – 6.12	0.442	0.78	0.24 – 2.53	0.680
N_{Trial}	16					
Observations	192					

```r
#Define the model with no choice as the reference category
modelMFSrefMalelow = mblogit(formula = choice.lowref ~ Sex.Mref,
                                  random = ~1|Trial,
                                  data = choiceMFS)

## Warning: Inner iterations did not coverge - nlminb message: false converge
nce
## (8)

##
## Iteration 1 - deviance = 270.2474 - criterion = 0.6597269

## Warning: Inner iterations did not coverge - nlminb message: false converge
nce
## (8)

##
## Iteration 2 - deviance = 262.2616 - criterion = 0.04104736

## Warning: Inner iterations did not coverge - nlminb message: false converge
nce
## (8)

##
## Iteration 3 - deviance = 260.1796 - criterion = 0.005949847

## Warning: Inner iterations did not coverge - nlminb message: false converge
nce
## (8)

##
## Iteration 4 - deviance = 259.3251 - criterion = 0.0002864956

## Warning: Inner iterations did not coverge - nlminb message: false converge
nce
## (8)

##
## Iteration 5 - deviance = 259.1496 - criterion = 7.851132e-08

## Warning: Inner iterations did not coverge - nlminb message: false converge
nce
## (8)

##
## Iteration 6 - deviance = 259.1462 - criterion = 6.210225e-15
## converged

#Call results
summary(modelMFSrefMalelow)

##
## Call:
```

```
## mblogit(formula = choice.lowref ~ Sex.Mref, data = choiceMFS,
##     random = ~1 | Trial)
##
## Equation for 1 - no choice vs 0 - low:
##              Estimate Std. Error z value Pr(>|z|)
## (Intercept)   -0.6466     0.5225  -1.238    0.216
## Sex.Mref2) F   0.6436     0.8307   0.775    0.438
## Sex.Mref3) S  -0.4721     0.6618  -0.713    0.476
##
## Equation for 2 - high vs 0 - low:
##              Estimate Std. Error z value Pr(>|z|)
## (Intercept)    1.5472     0.4313   3.587 0.000334 ***
## Sex.Mref2) F   1.2927     0.6354   2.034 0.041917 *
## Sex.Mref3) S  -0.7310     0.4643  -1.575 0.115367
## ---
## Signif. codes:  0 '***' 0.001 '**' 0.01 '*' 0.05 '.' 0.1 ' ' 1
##
## (Co-)Variances:
## Grouping level: Trial
##                 Estimate          Std.Err.
## 1 - no choice~1  0.4127           0.05487
## 2 - high~1      -0.2172  0.9602   0.13627 0.37961
##
## Approximate residual deviance: 259.1
## Number of Fisher scoring iterations:  6
## Number of observations
##   Groups by Trial: 16
##   Individual observations:  192

plot_model(modelMFSrefMalelow)
```

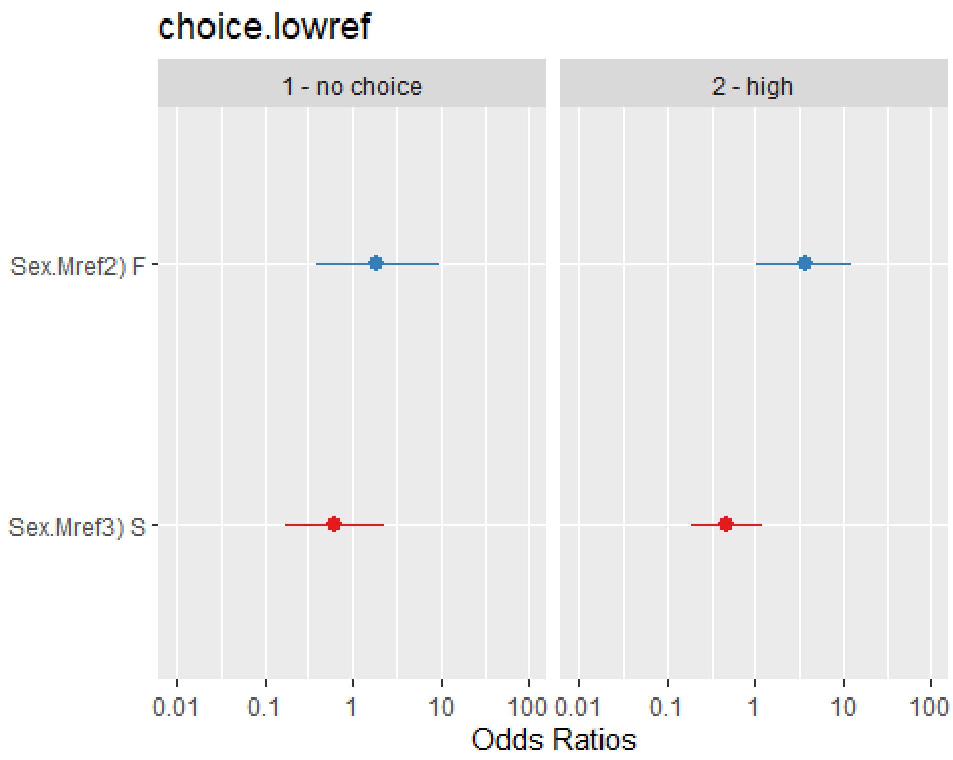


```
tab_model(modelMFSrefMalelow)
```

	choice.lowref: 1 - no choice			choice.lowref: 2 - high		
Predictors	*Odds Ratios*	*CI*	*p*	*Odds Ratios*	*CI*	*p*
(Intercept)	0.52	0.19 – 1.46	0.216	4.70	2.01 – 10.97	**<0.001**
Sex.Mref2) F	1.90	0.37 – 9.75	0.438	3.64	1.04 – 12.71	**0.042**
Sex.Mref3) S	0.62	0.17 – 2.29	0.476	0.48	0.19 – 1.20	0.115
N_{Trial}	16					
Observations	192					

```
#CHOICE FOR ADULTS (MALE AND FEMALE) ALL ROUNDS
#Read .csv file
choiceMF = read.csv("choiceMF.csv")

#Check variable status
summary(choiceMF)

##       Trial            Age                 Sex                 Round
##  Min.   : 1.00   Length:192         Length:192         Length:192
```

```
##  1st Qu.: 4.75   Class :character   Class :character   Class :character
##  Median : 8.50   Mode  :character   Mode  :character   Mode  :character
##  Mean   : 8.50
##  3rd Qu.:12.25
##  Max.   :16.00
##  choice.highref     choice.lowref      choice.nochoiceref
##  Length:192         Length:192         Length:192
##  Class :character   Class :character   Class :character
##  Mode  :character   Mode  :character   Mode  :character
##
##
##
```

```
#Reassign variable status
choiceMF$choice.nochoiceref = as.factor(choiceMF$choice.nochoiceref)
choiceMF$choice.lowref = as.factor(choiceMF$choice.lowref)
choiceMF$choice.highref = as.factor(choiceMF$choice.highref)
choiceMF$Round = as.factor(choiceMF$Round)
choiceMF$Age = as.factor(choiceMF$Age)
choiceMF$Sex = as.factor(choiceMF$Sex)
choiceMF$Trial = as.factor(choiceMF$Trial)

#Recheck variable status
summary(choiceMF)
```

```
##      Trial        Age       Sex                                    Round
##  1      : 12   Adult:192   F:96   2) Pair 12HR                        :32
##  2      : 12               M:96   3) Pair 24HR/Subordinate Added 0HR  :32
##  3      : 12                      4) Subordinate 12HR                 :32
##  4      : 12                      5) Subordinate 24HR/Coral Change 0HR:32
##  5      : 12                      6) Change 12HR                      :32
##  6      : 12                      7) Change 24HR                      :32
##  (Other):120
##    choice.highref       choice.lowref      choice.nochoiceref
##  high     :142    -1 - low      : 27    0 - no choice: 23
##  low      : 27    0 - no choice: 23    1 - low      : 27
##  no choice: 23    1 - high     :142    2 - high     :142
##
##
##
##
```

```
#Define the model with no choice as the reference category
modelMFrefnochoice = mblogit(formula = choice.nochoiceref~ Round,
                             random = ~1|Trial,
                             data = choiceMF)
```

```
## Warning: Inner iterations did not coverge - nlminb message: false converge
nce
## (8)
```

```
##
## Iteration 1 - deviance = 254.3332 - criterion = 0.6653286

## Warning: Inner iterations did not coverge - nlminb message: false converge
nce
## (8)

##
## Iteration 2 - deviance = 241.8937 - criterion = 0.05856489

## Warning: Inner iterations did not coverge - nlminb message: false converge
nce
## (8)

##
## Iteration 3 - deviance = 237.694 - criterion = 0.01056754

## Warning: Inner iterations did not coverge - nlminb message: false converge
nce
## (8)

##
## Iteration 4 - deviance = 236.0015 - criterion = 0.0005651547

## Warning: Inner iterations did not coverge - nlminb message: false converge
nce
## (8)

##
## Iteration 5 - deviance = 235.3633 - criterion = 0.0009867686

## Warning: Inner iterations did not coverge - nlminb message: false converge
nce
## (8)

##
## Iteration 6 - deviance = 235.2547 - criterion = 3.454551e-07

## Warning: Inner iterations did not coverge - nlminb message: false converge
nce
## (8)

##
## Iteration 7 - deviance = 235.2506 - criterion = 7.984618e-14
## converged

#Call results
summary(modelMFrefnochoice)

##
## Call:
## mblogit(formula = choice.nochoiceref ~ Round, data = choiceMF,
##     random = ~1 | Trial)
```

```
##
## Equation for 1 - low vs 0 - no choice:
##                                             Estimate Std. Error z value Pr(>
|z|)
## (Intercept)                                  -0.6289     0.6369  -0.987    0
.323
## Round3) Pair 24HR/Subordinate Added 0HR       0.9344     0.9249   1.010    0
.312
## Round4) Subordinate 12HR                     -0.1551     0.9706  -0.160    0
.873
## Round5) Subordinate 24HR/Coral Change 0HR     0.5256     1.0924   0.481    0
.630
## Round6) Change 12HR                           0.2186     0.8692   0.252    0
.801
## Round7) Change 24HR                           1.5366     1.2686   1.211    0
.226
##
## Equation for 2 - high vs 0 - no choice:
##                                             Estimate Std. Error z value Pr(>
|z|)
## (Intercept)                                   0.7979     0.5607   1.423   0.
1547
## Round3) Pair 24HR/Subordinate Added 0HR       1.4005     0.7945   1.763   0.
0779
## Round4) Subordinate 12HR                      1.2909     0.7397   1.745   0.
0810
## Round5) Subordinate 24HR/Coral Change 0HR     2.1313     0.8941   2.384   0.
0171
## Round6) Change 12HR                           0.8181     0.7053   1.160   0.
2460
## Round7) Change 24HR                           2.8186     1.1403   2.472   0.
0134
##
## (Intercept)
## Round3) Pair 24HR/Subordinate Added 0HR   .
## Round4) Subordinate 12HR                  .
## Round5) Subordinate 24HR/Coral Change 0HR *
## Round6) Change 12HR
## Round7) Change 24HR                       *
## ---
## Signif. codes:  0 '***' 0.001 '**' 0.01 '*' 0.05 '.' 0.1 ' ' 1
##
## (Co-)Variances:
## Grouping level: Trial
##             Estimate          Std.Err.
## 1 - low~1   1.0014           0.7713
## 2 - high~1 -0.3376  1.5538   1.2222 2.8237
##
## Approximate residual deviance: 235.3
## Number of Fisher scoring iterations:  7
```

```
## Number of observations
##   Groups by Trial: 16
##   Individual observations:  192
```

```
plot_model(modelMFrefnochoice)
```

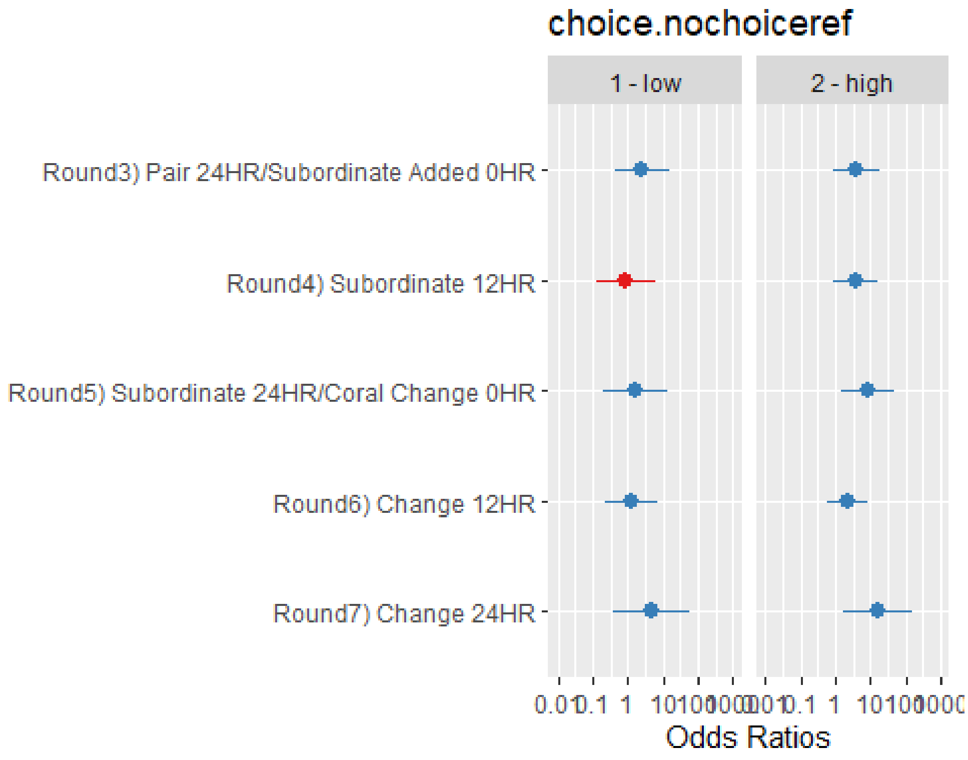


```
tab_model(modelMFrefnochoice)
```

	choice.nochoiceref: 1 - low			choice.nochoiceref: 2 - high		
Predictors	*Odds Ratios*	*CI*	*p*	*Odds Ratios*	*CI*	*p*
(Intercept)	0.53	0.15 – 1.87	0.323	2.22	0.74 – 6.69	0.155
Round3) Pair 24HR/Subordinate Added 0HR	2.55	0.41 – 15.69	0.312	4.06	0.85 – 19.35	0.078
Round4) Subordinate 12HR	0.86	0.13 – 5.77	0.873	3.64	0.85 – 15.57	0.081
Round5) Subordinate 24HR/Coral Change 0HR	1.69	0.20 – 14.49	0.630	8.43	1.45 – 48.88	**0.017**
Round6) Change 12HR	1.24	0.23 – 6.87	0.801	2.27	0.57 – 9.07	0.246
Round7) Change 24HR	4.65	0.38 – 56.33	0.226	16.75	1.78 – 157.72	**0.013**
N_{Trial}	16					
Observations	192					

```
#Define the model with High as the reference category
modelMFrefhigh = mblogit(formula = choice.highref ~ Round,
```

```
                         random = ~1|Trial,
                         data = choiceMF)

## Warning: Inner iterations did not coverge - nlminb message: false converge
nce
## (8)

##
## Iteration 1 - deviance = 254.4405 - criterion = 0.8213126

## Warning: Inner iterations did not coverge - nlminb message: false converge
nce
## (8)

##
## Iteration 2 - deviance = 241.2176 - criterion = 0.04848616

## Warning: Inner iterations did not coverge - nlminb message: false converge
nce
## (8)

##
## Iteration 3 - deviance = 236.7556 - criterion = 0.009910624

## Warning: Inner iterations did not coverge - nlminb message: false converge
nce
## (8)

##
## Iteration 4 - deviance = 234.6978 - criterion = 0.0009204383

## Warning: Inner iterations did not coverge - nlminb message: false converge
nce
## (8)

##
## Iteration 5 - deviance = 234.0307 - criterion = 1.050229e-06

## Warning: Inner iterations did not coverge - nlminb message: false converge
nce
## (8)

##
## Iteration 6 - deviance = 234.0066 - criterion = 1.29008e-12
## converged

#Call results
summary(modelMFrefhigh)

##
## Call:
## mblogit(formula = choice.highref ~ Round, data = choiceMF, random = ~1 |
##     Trial)
```

```
##
## Equation for low vs high:
##                                             Estimate Std. Error z value Pr(>
|z|)
## (Intercept)                                  -1.4307     0.7111  -2.012   0.
0442
## Round3) Pair 24HR/Subordinate Added 0HR      -0.4180     0.7784  -0.537   0.
5912
## Round4) Subordinate 12HR                     -1.4066     0.8798  -1.599   0.
1099
## Round5) Subordinate 24HR/Coral Change 0HR    -1.5459     0.8794  -1.758   0.
0788
## Round6) Change 12HR                          -0.5706     0.8008  -0.713   0.
4761
## Round7) Change 24HR                          -1.2127     0.8304  -1.460   0.
1442
##
## (Intercept)                                 *
## Round3) Pair 24HR/Subordinate Added 0HR
## Round4) Subordinate 12HR
## Round5) Subordinate 24HR/Coral Change 0HR .
## Round6) Change 12HR
## Round7) Change 24HR
##
## Equation for no choice vs high:
##                                             Estimate Std. Error z value Pr(>
|z|)
## (Intercept)                                  -0.8964     0.5928  -1.512   0.
1305
## Round3) Pair 24HR/Subordinate Added 0HR      -1.4371     0.8051  -1.785   0.
0743
## Round4) Subordinate 12HR                     -1.2898     0.7506  -1.718   0.
0857
## Round5) Subordinate 24HR/Coral Change 0HR    -2.1455     0.9043  -2.373   0.
0177
## Round6) Change 12HR                          -0.8303     0.7162  -1.159   0.
2463
## Round7) Change 24HR                          -2.8499     1.1488  -2.481   0.
0131
##
## (Intercept)
## Round3) Pair 24HR/Subordinate Added 0HR     .
## Round4) Subordinate 12HR                    .
## Round5) Subordinate 24HR/Coral Change 0HR *
## Round6) Change 12HR
## Round7) Change 24HR                         *
## ---
## Signif. codes:  0 '***' 0.001 '**' 0.01 '*' 0.05 '.' 0.1 ' ' 1
##
## (Co-)Variances:
```

```
## Grouping level: Trial
##              Estimate      Std.Err.
## low~1        2.823         2.3004
## no choice~1 1.322 1.954   1.6921 0.3575
##
## Approximate residual deviance: 234
## Number of Fisher scoring iterations:  6
## Number of observations
##   Groups by Trial: 16
##   Individual observations:  192
```

```
plot_model(modelMFrefhigh)
```

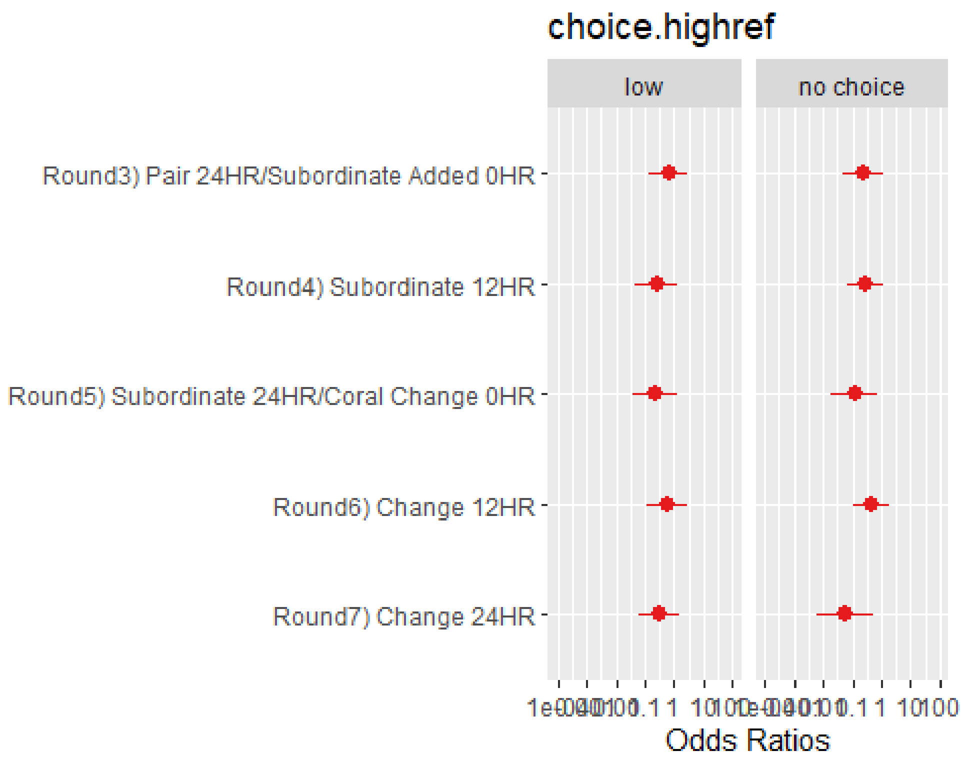


```
tab_model(modelMFrefhigh)
```

	choice.highref: low			choice.highref: no choice		
Predictors	*Odds Ratios*	*CI*	*p*	*Odds Ratios*	*CI*	*p*
(Intercept)	0.24	0.06 – 0.97	**0.044**	0.41	0.13 – 1.31	0.131
Round3) Pair 24HR/Subordinate Added 0HR	0.66	0.14 – 3.04	0.591	0.24	0.05 – 1.16	0.074
Round4) Subordinate 12HR	0.24	0.04 – 1.38	0.110	0.28	0.06 – 1.20	0.086
Round5) Subordinate 24HR/Coral Change 0HR	0.21	0.04 – 1.20	0.079	0.12	0.02 – 0.69	**0.018**
Round6) Change 12HR	0.57	0.12 – 2.73	0.476	0.44	0.11 – 1.78	0.246
Round7) Change 24HR	0.30	0.06 – 1.52	0.144	0.06	0.01 – 0.55	**0.013**
N_{Trial}	16					
Observations	192					

```
#CHOICE FOR SUBORDINATES ROUNDS 2-6 (FROM SUBORDINATE ADDITION)
#Read .csv file
choiceS = read.csv("choiceS.csv")

#Check variable status
summary(choiceS)

##      Trial           Age                Sex                Round          
##  Min.   : 1.00   Length:64          Length:64          Length:64         
##  1st Qu.: 4.75   Class :character   Class :character   Class :character  
##  Median : 8.50   Mode  :character   Mode  :character   Mode  :character  
##  Mean   : 8.50                                                           
##  3rd Qu.:12.25                                                           
##  Max.   :16.00                                                           
##  choice.highref     choice.nochoiceref choice.lowref     
##  Length:64          Length:64          Length:64         
##  Class :character   Class :character   Class :character  
##  Mode  :character   Mode  :character   Mode  :character  
## 
## 
## 

#Reassign variable status
choiceS$choice.nochoiceref = as.factor(choiceS$choice.nochoiceref)
choiceS$choice.highref = as.factor(choiceS$choice.highref)
choiceS$choice.lowref = as.factor(choiceS$choice.lowref)
choiceS$Round = as.factor(choiceS$Round)
choiceS$Age = as.factor(choiceS$Age)
choiceS$Sex = as.factor(choiceS$Sex)
choiceS$Trial = as.factor(choiceS$Trial)
```

```r
#Recheck variable status
summary(choiceS)
```

```
##      Trial            Age      Sex                                       Rou
nd
##  1      : 4   Subordinate:64   S:64   3) Subordinate 12HR
:16
##  2      : 4                           4) Subordinate 24HR/Coral Change 0HR
:16
##  3      : 4                           5) Change 12HR
:16
##  4      : 4                           6) Change 24HR
:16
##  5      : 4
##  6      : 4
##  (Other):40
##    choice.highref     choice.nochoiceref       choice.lowref
##  high      :39     0 - no choice: 7       -1 - low      :18
##  low       :18     1 - low      :18       0 - no choice: 7
##  no choice: 7      2 - high     :39       1 - high      :39
##
##
##
##
```

```r
#Define the model with no choice as the reference category
modelSrefnochoice = mblogit(formula = choice.nochoiceref ~ Round,
                            random = ~1|Trial,
                            data = choiceS)
```

```
## Warning: Inner iterations did not coverge - nlminb message: false converge
nce
## (8)

##
## Iteration 1 - deviance = 108.3278 - criterion = 0.8167445

## Warning: Inner iterations did not coverge - nlminb message: false converge
nce
## (8)

##
## Iteration 2 - deviance = 107.6659 - criterion = 0.02941433

## Warning: Inner iterations did not coverge - nlminb message: false converge
nce
## (8)

##
## Iteration 3 - deviance = 107.6566 - criterion = 0.0008178464
```

```
## Warning: Inner iterations did not coverge - nlminb message: false converge
nce
## (8)

##
## Iteration 4 - deviance = 107.6387 - criterion = 7.349965e-07

## Warning: Inner iterations did not coverge - nlminb message: false converge
nce
## (8)

##
## Iteration 5 - deviance = 107.6383 - criterion = 5.568472e-13
## converged
```

```
#Call results
summary(modelSrefnochoice)
```

```
##
## Call:
## mblogit(formula = choice.nochoiceref ~ Round, data = choiceS,
##     random = ~1 | Trial)
##
## Equation for 1 - low vs 0 - no choice:
##                                           Estimate Std. Error z value Pr(>
|z|)
## (Intercept)                                 0.3515     0.9293   0.378    0
.705
## Round4) Subordinate 24HR/Coral Change 0HR   0.9842     1.4457   0.681    0
.496
## Round5) Change 12HR                         0.1213     1.1720   0.103    0
.918
## Round6) Change 24HR                         1.4021     1.4167   0.990    0
.322
##
## Equation for 2 - high vs 0 - no choice:
##                                           Estimate Std. Error z value Pr(>
|z|)
## (Intercept)                                 1.7427     0.8089   2.155   0.
0312
## Round4) Subordinate 24HR/Coral Change 0HR   0.6903     1.3130   0.526   0.
5991
## Round5) Change 12HR                        -0.7886     1.0438  -0.755   0.
4500
## Round6) Change 24HR                         0.4433     1.3203   0.336   0.
7371
##
## (Intercept)                               *
## Round4) Subordinate 24HR/Coral Change 0HR
## Round5) Change 12HR
## Round6) Change 24HR
```

```
## ---
## Signif. codes:  0 '***' 0.001 '**' 0.01 '*' 0.05 '.' 0.1 ' ' 1
##
## (Co-)Variances:
## Grouping level: Trial
##              Estimate            Std.Err.
## 1 - low~1    0.36754             0.01421
## 2 - high~1 -0.02873  0.66013    0.02721 0.10094
##
## Approximate residual deviance: 107.6
## Number of Fisher scoring iterations:  5
## Number of observations
##   Groups by Trial: 16
##   Individual observations:  64
```

```
plot_model(modelSrefnochoice)
```

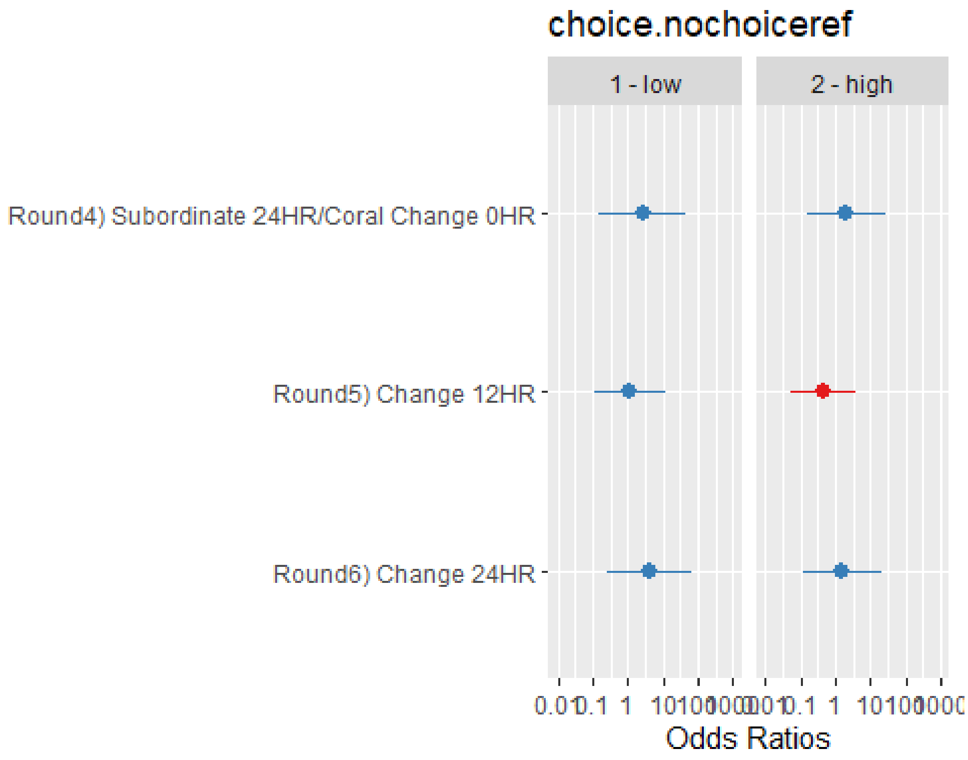


```
tab_model(modelSrefnochoice)
```

Predictors	choice.nochoiceref: 1 - low<br>*Odds Ratios*	*CI*	*p*	choice.nochoiceref: 2 - high<br>*Odds Ratios*	*CI*	*p*
(Intercept)	1.42	0.23 – 8.95	0.705	5.71	1.15 – 28.35	**0.031**
Round4) Subordinate 24HR/Coral Change 0HR	2.68	0.15 – 46.87	0.496	1.99	0.15 – 26.86	0.599
Round5) Change 12HR	1.13	0.11 – 11.50	0.918	0.45	0.06 – 3.59	0.450
Round6) Change 24HR	4.06	0.25 – 67.21	0.322	1.56	0.11 – 21.28	0.737
N_{Trial}	16					
Observations	64					

```
#Define the model with High as the reference category
modelSreflow = mblogit(formula = choice.lowref ~ Round,
                        random = ~1|Trial,
                        data = choiceS)

## Warning: Inner iterations did not coverge - nlminb message: false converge
nce
## (8)

##
## Iteration 1 - deviance = 106.9057 - criterion = 0.628672

## Warning: Inner iterations did not coverge - nlminb message: false converge
nce
## (8)

##
## Iteration 2 - deviance = 104.2482 - criterion = 0.08853423

## Warning: Inner iterations did not coverge - nlminb message: false converge
nce
## (8)

##
## Iteration 3 - deviance = 102.8816 - criterion = 0.007482457

## Warning: Inner iterations did not coverge - nlminb message: false converge
nce
## (8)

##
## Iteration 4 - deviance = 102.4361 - criterion = 0.00172834

## Warning: Inner iterations did not coverge - nlminb message: false converge
nce
## (8)
```

```
##
## Iteration 5 - deviance = 102.1338 - criterion = 8.980019e-07

## Warning: Inner iterations did not coverge - nlminb message: false converge
nce
## (8)

##
## Iteration 6 - deviance = 102.1274 - criterion = 2.980933e-13
## converged
```

```
#Call results
summary(modelSreflow)
```

```
##
## Call:
## mblogit(formula = choice.lowref ~ Round, data = choiceS, random = ~1 |
##     Trial)
##
## Equation for 0 - no choice vs -1 - low:
##                                            Estimate Std. Error z value Pr(>
|z|)
## (Intercept)                                -0.79953    0.99566  -0.803    0
.422
## Round4) Subordinate 24HR/Coral Change 0HR -1.04539    1.48853  -0.702    0
.482
## Round5) Change 12HR                         0.04259    1.21777   0.035    0
.972
## Round6) Change 24HR                        -1.37606    1.45778  -0.944    0
.345
##
## Equation for 1 - high vs -1 - low:
##                                            Estimate Std. Error z value Pr(>
|z|)
## (Intercept)                                  1.3256     0.7457   1.777   0.
0755
## Round4) Subordinate 24HR/Coral Change 0HR  -0.2486     0.9265  -0.268   0.
7884
## Round5) Change 12HR                         -0.9748     0.9115  -1.069   0.
2849
## Round6) Change 24HR                         -0.9430     0.8881  -1.062   0.
2883
##
## (Intercept)                                .
## Round4) Subordinate 24HR/Coral Change 0HR
## Round5) Change 12HR
## Round6) Change 24HR
## ---
## Signif. codes:  0 '***' 0.001 '**' 0.01 '*' 0.05 '.' 0.1 ' ' 1
##
## (Co-)Variances:
```

```
## Grouping level: Trial
##                    Estimate          Std.Err.
## 0 - no choice~1  0.9071             1.164
## 1 - high~1      -0.4753  1.3367    1.806 2.629
##
## Approximate residual deviance: 102.1
## Number of Fisher scoring iterations:  6
## Number of observations
##   Groups by Trial: 16
##   Individual observations:  64
```

```r
plot_model(modelSreflow)
```

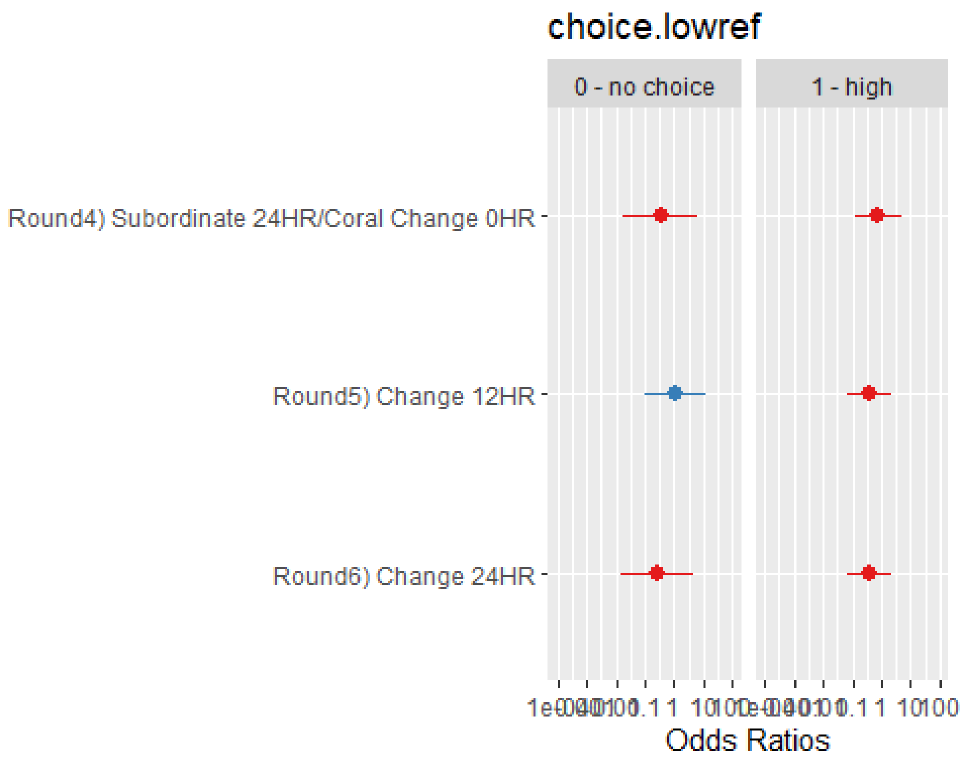


```r
tab_model(modelSreflow)
```

	choice.lowref: 0 - no choice			choice.lowref: 1 - high		
Predictors	*Odds Ratios*	*CI*	*p*	*Odds Ratios*	*CI*	*p*
(Intercept)	0.45	0.06 – 3.23	0.422	3.76	0.86 – 16.49	0.075
Round4) Subordinate 24HR/Coral Change 0HR	0.35	0.02 – 6.70	0.482	0.78	0.12 – 4.89	0.788
Round5) Change 12HR	1.04	0.09 – 11.64	0.972	0.38	0.06 – 2.29	0.285
Round6) Change 24HR	0.25	0.01 – 4.53	0.345	0.39	0.07 – 2.26	0.288
N_{Trial}	16					
Observations	64					

```
#Define the model with no choice as the reference category
modelSrefhigh = mblogit(formula = choice.highref ~ Round,
                              random = ~1|Trial,
                              data = choiceS)

## Warning: Inner iterations did not coverge - nlminb message: false converge
nce
## (8)

##
## Iteration 1 - deviance = 110.0097 - criterion = 0.8716433

## Warning: Inner iterations did not coverge - nlminb message: false converge
nce
## (8)

##
## Iteration 2 - deviance = 108.4588 - criterion = 0.01518287

## Warning: Inner iterations did not coverge - nlminb message: false converge
nce
## (8)

##
## Iteration 3 - deviance = 108.4002 - criterion = 0.0004259881

## Warning: Inner iterations did not coverge - nlminb message: false converge
nce
## (8)

##
## Iteration 4 - deviance = 108.3644 - criterion = 2.791728e-07

## Warning: Inner iterations did not coverge - nlminb message: false converge
nce
## (8)
```

```
##
## Iteration 5 - deviance = 108.3636 - criterion = 1.595901e-13
## converged
```

```
#Call results
summary(modelSrefhigh)
```

```
##
## Call:
## mblogit(formula = choice.highref ~ Round, data = choiceS, random = ~1 |
##     Trial)
##
## Equation for low vs high:
##                                        Estimate Std. Error z value Pr(>
|z|)
## (Intercept)                             -1.3154     0.6741  -1.951    0
.051
## Round4) Subordinate 24HR/Coral Change 0HR   0.2866     0.8821   0.325    0
.745
## Round5) Change 12HR                      0.8488     0.8729   0.972    0
.331
## Round6) Change 24HR                      0.9056     0.8455   1.071    0
.284
##
## (Intercept)                            .
## Round4) Subordinate 24HR/Coral Change 0HR
## Round5) Change 12HR
## Round6) Change 24HR
##
## Equation for no choice vs high:
##                                        Estimate Std. Error z value Pr(>
|z|)
## (Intercept)                             -1.7527     0.8011  -2.188   0.
0287
## Round4) Subordinate 24HR/Coral Change 0HR  -0.7016     1.3091  -0.536   0.
5920
## Round5) Change 12HR                      0.7524     1.0386   0.724   0.
4688
## Round6) Change 24HR                     -0.4860     1.3166  -0.369   0.
7120
##
## (Intercept)                            *
## Round4) Subordinate 24HR/Coral Change 0HR
## Round5) Change 12HR
## Round6) Change 24HR
## ---
## Signif. codes:  0 '***' 0.001 '**' 0.01 '*' 0.05 '.' 0.1 ' ' 1
##
## (Co-)Variances:
## Grouping level: Trial
```

```
##               Estimate        Std.Err.
## low~1         0.3730          0.04111
## no choice~1 0.2081 0.5367    0.05750 0.08528
##
## Approximate residual deviance: 108.4
## Number of Fisher scoring iterations:  5
## Number of observations
##   Groups by Trial: 16
##   Individual observations:  64

plot_model(modelSrefhigh)
```

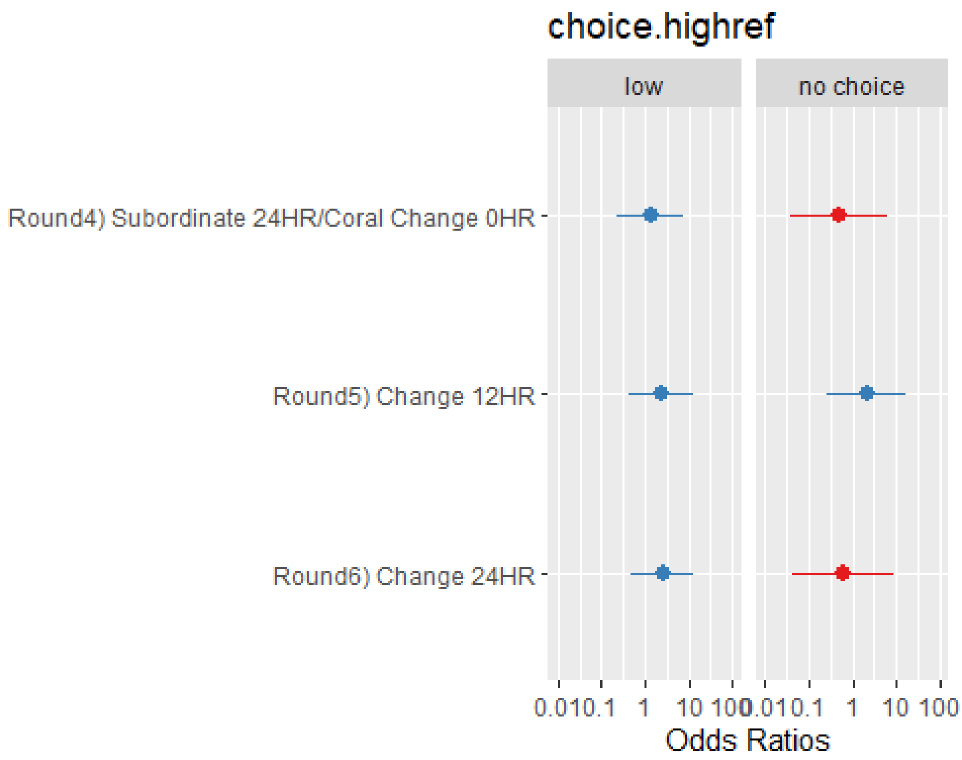


```
tab_model(modelSrefhigh)
```

	choice.highref: low			choice.highref: no choice		
Predictors	*Odds Ratios*	*CI*	*p*	*Odds Ratios*	*CI*	*p*
(Intercept)	0.27	0.07 – 1.02	0.051	0.17	0.04 – 0.85	**0.029**
Round4) Subordinate 24HR/Coral Change 0HR	1.33	0.23 – 7.64	0.745	0.50	0.04 – 6.63	0.592
Round5) Change 12HR	2.34	0.41 – 13.17	0.331	2.12	0.27 – 16.60	0.469
Round6) Change 24HR	2.47	0.46 – 13.20	0.284	0.62	0.05 – 8.34	0.712
N_{Trial}	16					
Observations	64					

```
#Set working directory
setwd("C:/Users/court/OneDrive/Desktop/OTI Binary Choice")

#Call packages
library(lme4)
library(lmerTest)
library(car)
library(mclogit)
library(nnet)
library(memisc)
library(sjPlot)
library(performance)

## Warning: package 'performance' was built under R version 4.4.2

#SWITCH GLM
#Read glm.csv
switchglmadult = read.csv("switch glm2adults.csv")

#Check status of variables
summary(switchglmadult)

##     Group.ID       Individual        Sex                 Age
##  Min.   : 1.00   Min.   : 1.0   Length:96          Length:96
##  1st Qu.: 4.75   1st Qu.:12.5   Class :character   Class :character
##  Median : 8.50   Median :24.0   Mode  :character   Mode  :character
##  Mean   : 8.50   Mean   :24.0
##  3rd Qu.:12.25   3rd Qu.:35.5
##  Max.   :16.00   Max.   :47.0
##      Round      Switches
##  Min.   :1   Min.   :0.0000
##  1st Qu.:1   1st Qu.:0.0000
##  Median :2   Median :0.0000
##  Mean   :2   Mean   :0.6458
##  3rd Qu.:3   3rd Qu.:1.0000
##  Max.   :3   Max.   :3.0000

#Reassign variables types
switchglmadult$Round = as.factor(switchglmadult$Round)
switchglmadult$Age = as.factor(switchglmadult$Age)
switchglmadult$Sex = as.factor(switchglmadult$Sex)
switchglmadult$Individual = as.factor(switchglmadult$Individual)
switchglmadult$Group.ID = as.factor(switchglmadult$Group.ID)
switchglmadult$Switches = as.numeric(switchglmadult$Switches)

#Recheck status of variables
summary(switchglmadult)
```

```
##     Group.ID    Individual Sex       Age      Round     Switches
##  1       : 6   1       : 3   F:48   Adult:96   1:32   Min.   :0.0000
##  2       : 6   2       : 3   M:48              2:32   1st Qu.:0.0000
##  3       : 6   4       : 3                     3:32   Median :0.0000
##  4       : 6   5       : 3                            Mean   :0.6458
##  5       : 6   7       : 3                            3rd Qu.:1.0000
##  6       : 6   8       : 3                            Max.   :3.0000
##  (Other):60   (Other):78

#GLMER for number of switches (Frequency per trial round)
model = glmer(Switches ~ Sex + Round +Sex*Round + (1|Group.ID), data = switch
glmadult, family = "poisson")

summary(model)

## Generalized linear mixed model fit by maximum likelihood (Laplace
##   Approximation) [glmerMod]
##  Family: poisson  ( log )
## Formula: Switches ~ Sex + Round + Sex * Round + (1 | Group.ID)
##    Data: switchglmadult
##
##      AIC      BIC   logLik deviance df.resid
##    195.5    213.4    -90.7    181.5       89
##
## Scaled residuals:
##     Min      1Q  Median      3Q     Max
## -0.9795 -0.5539 -0.4350  0.3618  3.0427
##
## Random effects:
##  Groups   Name        Variance Std.Dev.
##  Group.ID (Intercept) 0.2982   0.5461
## Number of obs: 96, groups:  Group.ID, 16
##
## Fixed effects:
##               Estimate Std. Error z value Pr(>|z|)
## (Intercept)   0.1685     0.2669   0.631  0.52785
## SexM         -0.3185     0.3286  -0.969  0.33245
## Round2       -1.2993     0.4606  -2.821  0.00479 **
## Round3       -1.2993     0.4606  -2.821  0.00479 **
## SexM:Round2  -0.0870     0.7243  -0.120  0.90440
## SexM:Round3   0.6061     0.6322   0.959  0.33766
## ---
## Signif. codes:  0 '***' 0.001 '**' 0.01 '*' 0.05 '.' 0.1 ' ' 1
##
## Correlation of Fixed Effects:
##             (Intr) SexM   Round2 Round3 SxM:R2
## SexM        -0.518
## Round2      -0.370  0.300
## Round3      -0.370  0.300  0.214
```

```
## SexM:Round2  0.235 -0.454 -0.636 -0.136
## SexM:Round3  0.269 -0.520 -0.156 -0.729  0.236
```

```r
anova(model)
```

```
## Analysis of Variance Table
##             npar  Sum Sq Mean Sq F value
## Sex            1  0.9747  0.9747  0.9747
## Round          2 18.6020  9.3010  9.3010
## Sex:Round      2  1.0137  0.5068  0.5068
```

```r
Anova(model, type = "III")
```

```
## Analysis of Deviance Table (Type III Wald chisquare tests)
##
## Response: Switches
##               Chisq Df Pr(>Chisq)
## (Intercept)  0.3985  1   0.527854
## Sex          0.9393  1   0.332447
## Round       13.1075  2   0.001425 **
## Sex:Round    1.0462  2   0.592680
## ---
## Signif. codes:  0 '***' 0.001 '**' 0.01 '*' 0.05 '.' 0.1 ' ' 1
```

```r
tab_model(model)
```

	**Switches**		
Predictors	*Incidence Rate Ratios*	*CI*	*p*
(Intercept)	1.18	0.70 – 2.00	0.528
Sex [M]	0.73	0.38 – 1.38	0.332
Round [2]	0.27	0.11 – 0.67	**0.005**
Round [3]	0.27	0.11 – 0.67	**0.005**
Sex [M] × Round [2]	0.92	0.22 – 3.79	0.904
Sex [M] × Round [3]	1.83	0.53 – 6.33	0.338
**Random Effects**			
σ^2	0.94		
$\tau_{00\ Group.ID}$	0.30		
ICC	0.24		
$N_{Group.ID}$	16		
Observations	96		
Marginal R^2 / Conditional R^2	0.224 / 0.412		

```r
check_overdispersion(model)
```

```
## # Overdispersion test
##
##        dispersion ratio =  0.770
##   Pearson's Chi-Squared = 68.538
##                 p-value =  0.947

## No overdispersion detected.
```

```r
check_collinearity(model)
```

```
## # Check for Multicollinearity
##
## Low Correlation
##
##   Term  VIF   VIF 95% CI Increased SE Tolerance Tolerance 95% CI
##    Sex 1.66 [1.35, 2.25]         1.29      0.60     [0.44, 0.74]
##  Round 3.68 [2.74, 5.14]         1.92      0.27     [0.19, 0.36]
##
## Moderate Correlation
##
##       Term  VIF   VIF 95% CI Increased SE Tolerance Tolerance 95% CI
##  Sex:Round 5.11 [3.73, 7.19]         2.26      0.20     [0.14, 0.27]
```

```r
check_distribution(model)
```

```
## # Distribution of Model Family
##
## Predicted Distribution of Residuals
##
##  Distribution Probability
##        normal         41%
##       tweedie         22%
##        cauchy          9%
##
## Predicted Distribution of Response
##
##                 Distribution Probability
##        poisson (zero-infl.)         50%
##  neg. binomial (zero-infl.)         28%
##                beta-binomial         12%
```

```r
check_homogeneity(model)
```

```
## OK: There is not clear evidence for different variances across groups (Bar
tlett Test, p = 0.546).
```

```r
#GLMER testing negative binomial vs poisson
model.nb = glmer.nb(Switches ~ Sex + Round +Sex*Round + (1|Group.ID), data =
switchglmadult,)
```

```
## Warning in theta.ml(Y, mu, weights = object@resp$weights, limit = limit, :
## iteration limit reached
```

```r
summary(model.nb)

## Generalized linear mixed model fit by maximum likelihood (Laplace
##   Approximation) [glmerMod]
##  Family: Negative Binomial(33615.34)  ( log )
## Formula: Switches ~ Sex + Round + Sex * Round + (1 | Group.ID)
##    Data: switchglmadult
##
##      AIC      BIC   logLik deviance df.resid
##    197.5    218.0    -90.7    181.5       88
##
## Scaled residuals:
##     Min      1Q  Median      3Q     Max
## -0.9795 -0.5539 -0.4350  0.3618  3.0427
##
## Random effects:
##  Groups   Name        Variance Std.Dev.
##  Group.ID (Intercept) 0.2982   0.5461
## Number of obs: 96, groups:  Group.ID, 16
##
## Fixed effects:
##             Estimate Std. Error z value Pr(>|z|)
## (Intercept)  0.16853    0.26318   0.640  0.52194
## SexM        -0.31846    0.32143  -0.991  0.32180
## Round2      -1.29928    0.44567  -2.915  0.00355 **
## Round3      -1.29928    0.44559  -2.916  0.00355 **
## SexM:Round2 -0.08699    0.69374  -0.125  0.90021
## SexM:Round3  0.60614    0.60803   0.997  0.31881
## ---
## Signif. codes:  0 '***' 0.001 '**' 0.01 '*' 0.05 '.' 0.1 ' ' 1
##
## Correlation of Fixed Effects:
##             (Intr) SexM   Round2 Round3 SxM:R2
## SexM        -0.506
## Round2      -0.357  0.276
## Round3      -0.353  0.276  0.202
## SexM:Round2  0.221 -0.438 -0.613 -0.121
## SexM:Round3  0.249 -0.505 -0.138 -0.711  0.225

anova(model.nb)

## Analysis of Variance Table
##           npar  Sum Sq Mean Sq F value
## Sex          1  0.9747  0.9747  0.9747
## Round        2 18.6021  9.3010  9.3010
## Sex:Round    2  1.0137  0.5069  0.5069

Anova(model.nb, type = "III")

## Analysis of Deviance Table (Type III Wald chisquare tests)
##
```

```
## Response: Switches
##                Chisq Df Pr(>Chisq)
## (Intercept)  0.4101  1  0.5219434
## Sex          0.9816  1  0.3218032
## Round       14.1454  2  0.0008479 ***
## Sex:Round    1.1230  2  0.5703670
## ---
## Signif. codes:  0 '***' 0.001 '**' 0.01 '*' 0.05 '.' 0.1 ' ' 1
```

```
#GLMER with backwards stepwise removal of interaction term
modelbsr = glmer(Switches ~ Sex + Round + (1|Group.ID), data = switchglmadult
, family = "poisson")

summary(modelbsr)
```

```
## Generalized linear mixed model fit by maximum likelihood (Laplace
##   Approximation) [glmerMod]
##  Family: poisson  ( log )
## Formula: Switches ~ Sex + Round + (1 | Group.ID)
##    Data: switchglmadult
##
##      AIC      BIC   logLik deviance df.resid
##    192.6    205.4    -91.3    182.6       91
##
## Scaled residuals:
##     Min      1Q  Median      3Q     Max
## -0.9533 -0.5577 -0.4464  0.2908  3.2836
##
## Random effects:
##  Groups   Name        Variance Std.Dev.
##  Group.ID (Intercept) 0.2982   0.5461
## Number of obs: 96, groups:  Group.ID, 16
##
## Fixed effects:
##             Estimate Std. Error z value Pr(>|z|)
## (Intercept)   0.1143     0.2557   0.447 0.654846
## SexM         -0.1941     0.2552  -0.761 0.446793
## Round2       -1.3350     0.3554  -3.756 0.000172 ***
## Round3       -0.9985     0.3126  -3.194 0.001404 **
## ---
## Signif. codes:  0 '***' 0.001 '**' 0.01 '*' 0.05 '.' 0.1 ' ' 1
##
## Correlation of Fixed Effects:
##        (Intr) SexM   Round2
## SexM   -0.451
## Round2 -0.290  0.000
## Round3 -0.329  0.000  0.237
```

```
anova(modelbsr)
```

```
## Analysis of Variance Table
##       npar  Sum Sq Mean Sq F value
## Sex      1  0.5608  0.5608  0.5608
## Round    2 19.1208  9.5604  9.5604
```

```
Anova(modelbsr, type = "III")
```

```
## Analysis of Deviance Table (Type III Wald chisquare tests)
##
## Response: Switches
##               Chisq Df Pr(>Chisq)
## (Intercept)  0.1998  1     0.6548
## Sex          0.5788  1     0.4468
## Round       19.7347  2  5.184e-05 ***
## ---
## Signif. codes:  0 '***' 0.001 '**' 0.01 '*' 0.05 '.' 0.1 ' ' 1
```

```
check_overdispersion(modelbsr)
```

```
## # Overdispersion test
##
##        dispersion ratio =  0.784
##   Pearson's Chi-Squared = 71.354
##                 p-value =  0.937
```

```
## No overdispersion detected.
```

```
check_collinearity(modelbsr)
```

```
## # Check for Multicollinearity
##
## Low Correlation
##
##   Term  VIF  VIF 95% CI Increased SE Tolerance Tolerance 95% CI
##    Sex 1.00 [1.00, Inf]         1.00      1.00     [0.00, 1.00]
##  Round 1.00 [1.00, Inf]         1.00      1.00     [0.00, 1.00]
```

```
check_distribution(modelbsr)
```

```
## # Distribution of Model Family
##
## Predicted Distribution of Residuals
##
##  Distribution Probability
##        normal         41%
##       tweedie         22%
##        cauchy          9%
##
## Predicted Distribution of Response
##
##                Distribution Probability
##        poisson (zero-infl.)         50%
```

```
##  neg. binomial (zero-infl.)         28%
##                beta-binomial         12%

check_homogeneity(modelbsr)

## OK: There is not clear evidence for different variances across groups (Bar
tlett Test, p = 0.546).

#with subs to compare
#Read glm.csv
switchglmwsub = read.csv("switch glm2r23.csv")

#Check status of variables
summary(switchglmwsub)

##     Group.ID        Individual          Sex                  Age
##  Min.   : 1.00   Min.   : 1.00   Length:96          Length:96
##  1st Qu.: 4.75   1st Qu.:12.75   Class :character   Class :character
##  Median : 8.50   Median :24.50   Mode  :character   Mode  :character
##  Mean   : 8.50   Mean   :24.50
##  3rd Qu.:12.25   3rd Qu.:36.25
##  Max.   :16.00   Max.   :48.00
##      Round          Switches
##  Min.   :2.0   Min.   :0.0000
##  1st Qu.:2.0   1st Qu.:0.0000
##  Median :2.5   Median :0.0000
##  Mean   :2.5   Mean   :0.5833
##  3rd Qu.:3.0   3rd Qu.:1.0000
##  Max.   :3.0   Max.   :3.0000

#Reassign variables types
switchglmwsub$Round = as.factor(switchglmwsub$Round)
switchglmwsub$Age = as.factor(switchglmwsub$Age)
switchglmwsub$Sex = as.factor(switchglmwsub$Sex)
switchglmwsub$Individual = as.factor(switchglmwsub$Individual)
switchglmwsub$Group.ID = as.factor(switchglmwsub$Group.ID)
switchglmwsub$Switches = as.numeric(switchglmwsub$Switches)

#Recheck status of variables
summary(switchglmwsub)

##     Group.ID     Individual  Sex              Age     Round      Switches
##  1      : 6    1      : 2    F:32   Adult      :64   2:48   Min.   :0.0000
##  2      : 6    2      : 2    M:32   Subordinate:32   3:48   1st Qu.:0.0000
##  3      : 6    3      : 2    S:32                           Median :0.0000
##  4      : 6    4      : 2                                   Mean   :0.5833
##  5      : 6    5      : 2                                   3rd Qu.:1.0000
##  6      : 6    6      : 2                                   Max.   :3.0000
##  (Other):60   (Other):84
```

```
#GLMER for number of switches (Frequency per trial round)
model2 = glmer(Switches ~ Sex + Round +Sex*Round + (1|Group.ID), data = switc
hglmwsub, family = "poisson")

summary(model2)

## Generalized linear mixed model fit by maximum likelihood (Laplace
##   Approximation) [glmerMod]
##  Family: poisson  ( log )
## Formula: Switches ~ Sex + Round + Sex * Round + (1 | Group.ID)
##    Data: switchglmwsub
##
##      AIC      BIC   logLik deviance df.resid
##    197.0    215.0    -91.5    183.0       89
##
## Scaled residuals:
##     Min      1Q  Median      3Q     Max
## -1.0870 -0.6303 -0.5068  0.7184  3.7309
##
## Random effects:
##  Groups   Name        Variance Std.Dev.
##  Group.ID (Intercept) 0.06583  0.2566
## Number of obs: 96, groups:  Group.ID, 16
##
## Fixed effects:
##               Estimate Std. Error z value Pr(>|z|)
## (Intercept) -1.013e+00  4.168e-01  -2.432   0.0150 *
## SexM        -4.055e-01  6.439e-01  -0.630   0.5289
## SexS         1.041e+00  4.737e-01   2.199   0.0279 *
## Round3       1.172e-05  5.759e-01   0.000   1.0000
## SexM:Round3  6.931e-01  8.396e-01   0.826   0.4090
## SexS:Round3 -1.252e-01  6.757e-01  -0.185   0.8530
## ---
## Signif. codes:  0 '***' 0.001 '**' 0.01 '*' 0.05 '.' 0.1 ' ' 1
##
## Correlation of Fixed Effects:
##             (Intr) SexM   SexS   Round3 SxM:R3
## SexM        -0.618
## SexS        -0.840  0.544
## Round3      -0.691  0.447  0.608
## SexM:Round3  0.474 -0.767 -0.417 -0.686
## SexS:Round3  0.589 -0.381 -0.701 -0.852  0.585

anova(model2)

## Analysis of Variance Table
##           npar  Sum Sq Mean Sq F value
## Sex          2 12.2473  6.1237  6.1237
## Round        1  0.0520  0.0520  0.0520
## Sex:Round    2  1.3242  0.6621  0.6621
```

```r
Anova(model2, type = "III")

## Analysis of Deviance Table (Type III Wald chisquare tests)
##
## Response: Switches
##              Chisq Df Pr(>Chisq)
## (Intercept) 5.9125  1   0.015034 *
## Sex         9.5635  2   0.008381 **
## Round       0.0000  1   0.999984
## Sex:Round   1.3596  2   0.506709
## ---
## Signif. codes:  0 '***' 0.001 '**' 0.01 '*' 0.05 '.' 0.1 ' ' 1

tab_model(model2)
```

	Switches		
Predictors	*Incidence Rate Ratios*	*CI*	*p*
(Intercept)	0.36	0.16 – 0.82	**0.015**
Sex [M]	0.67	0.19 – 2.36	0.529
Sex [S]	2.83	1.12 – 7.17	**0.028**
Round [3]	1.00	0.32 – 3.09	1.000
Sex [M] × Round [3]	2.00	0.39 – 10.37	0.409
Sex [S] × Round [3]	0.88	0.23 – 3.32	0.853
**Random Effects**			
σ^2	1.00		
τ_{00} Group.ID	0.07		
ICC	0.06		
N Group.ID	16		
Observations	96		
Marginal R^2 / Conditional R^2	0.203 / 0.252		

```r
check_overdispersion(model2)

## # Overdispersion test
##
##        dispersion ratio =  1.055
##   Pearson's Chi-Squared = 93.879
##                 p-value =  0.341

## No overdispersion detected.
```

```r
check_collinearity(model2)
```

```
## Model has interaction terms. VIFs might be inflated.
##   You may check multicollinearity among predictors of a model without
##   interaction terms.
```

```
## # Check for Multicollinearity
##
## Low Correlation
##
##   Term  VIF     VIF 95% CI Increased SE Tolerance Tolerance 95% CI
##  Round 4.66 [ 3.42,  6.54]         2.16      0.21     [0.15, 0.29]
##
## Moderate Correlation
##
##  Term  VIF     VIF 95% CI Increased SE Tolerance Tolerance 95% CI
##   Sex 5.45 [ 3.96,  7.67]         2.33      0.18     [0.13, 0.25]
##
## High Correlation
##
##       Term   VIF     VIF 95% CI Increased SE Tolerance Tolerance 95% CI
##  Sex:Round 15.12 [10.68, 21.62]         3.89      0.07     [0.05, 0.09]
```

```r
check_distribution(model2)
```

```
## # Distribution of Model Family
##
## Predicted Distribution of Residuals
##
##  Distribution Probability
##        normal         53%
##       tweedie         25%
##        cauchy          9%
##
## Predicted Distribution of Response
##
##          Distribution Probability
##              binomial         34%
##  poisson (zero-infl.)         34%
##         beta-binomial         16%
```

```r
check_homogeneity(model2)
```

```
## OK: There is not clear evidence for different variances across groups (Bar
tlett Test, p = 0.755).
```

```r
#GLMER with backwards stepwise removal of interaction term
model2bsr = glmer(Switches ~ Sex + Round + (1|Group.ID), data = switchglmwsub
, family = "poisson")

summary(model2bsr)
```

```
## Generalized linear mixed model fit by maximum likelihood (Laplace
##   Approximation) [glmerMod]
##  Family: poisson  ( log )
## Formula: Switches ~ Sex + Round + (1 | Group.ID)
##    Data: switchglmwsub
##
##      AIC      BIC   logLik deviance df.resid
##    194.4    207.2    -92.2    184.4       91
##
## Scaled residuals:
##     Min      1Q  Median      3Q     Max
## -1.0732 -0.6068 -0.5698  0.8089  3.8241
##
## Random effects:
##  Groups   Name        Variance Std.Dev.
##  Group.ID (Intercept) 0.06583  0.2566
## Number of obs: 96, groups:  Group.ID, 16
##
## Fixed effects:
##               Estimate Std. Error z value Pr(>|z|)
## (Intercept) -1.050e+00  3.315e-01  -3.167  0.00154 **
## SexM         1.726e-05  4.072e-01   0.000  0.99997
## SexS         9.808e-01  3.377e-01   2.905  0.00368 **
## Round3       7.146e-02  2.668e-01   0.268  0.78881
## ---
## Signif. codes:  0 '***' 0.001 '**' 0.01 '*' 0.05 '.' 0.1 ' ' 1
##
## Correlation of Fixed Effects:
##        (Intr) SexM   SexS
## SexM   -0.614
## SexS   -0.741  0.603
## Round3 -0.417  0.000  0.000
```

```
anova(model2bsr)
```

```
## Analysis of Variance Table
##       npar  Sum Sq Mean Sq F value
## Sex      2 12.9128  6.4564  6.4564
## Round    1  0.0699  0.0699  0.0699
```

```
Anova(model2bsr, type = "III")
```

```
## Analysis of Deviance Table (Type III Wald chisquare tests)
##
## Response: Switches
##               Chisq Df Pr(>Chisq)
## (Intercept) 10.0303  1   0.001540 **
## Sex         13.2584  2   0.001321 **
## Round        0.0717  1   0.788808
## ---
## Signif. codes:  0 '***' 0.001 '**' 0.01 '*' 0.05 '.' 0.1 ' ' 1
```

```r
check_overdispersion(model2bsr)

## # Overdispersion test
##
##        dispersion ratio =  1.043
##   Pearson's Chi-Squared = 94.934
##                 p-value =  0.368

## No overdispersion detected.

check_collinearity(model2bsr)

## # Check for Multicollinearity
##
## Low Correlation
##
##   Term  VIF  VIF 95% CI Increased SE Tolerance Tolerance 95% CI
##    Sex 1.00 [1.00, Inf]         1.00      1.00     [0.00, 1.00]
##  Round 1.00 [1.00, Inf]         1.00      1.00     [0.00, 1.00]

check_distribution(model2bsr)

## # Distribution of Model Family
##
## Predicted Distribution of Residuals
##
##  Distribution Probability
##        normal         50%
##       tweedie         28%
##        cauchy          9%
##
## Predicted Distribution of Response
##
##          Distribution Probability
##              binomial         34%
##  poisson (zero-infl.)         34%
##         beta-binomial         16%

check_homogeneity(model2bsr)

## OK: There is not clear evidence for different variances across groups (Bar
tlett Test, p = 0.755).

#only subs to see if influence of round
#Read glm.csv
switchglmsubo = read.csv("switch glm2r23sub.csv")

#Check status of variables
summary(switchglmsubo)

##     Group.ID       Individual        Sex                  Age
##  Min.   : 1.00   Min.   : 3.00   Length:32          Length:32
```

```
##  1st Qu.: 4.75   1st Qu.:14.25   Class :character   Class :character
##  Median : 8.50   Median :25.50   Mode  :character   Mode  :character
##  Mean   : 8.50   Mean   :25.50
##  3rd Qu.:12.25   3rd Qu.:36.75
##  Max.   :16.00   Max.   :48.00
##      Round         Switches
##  Min.   :2.0   Min.   :0
##  1st Qu.:2.0   1st Qu.:0
##  Median :2.5   Median :1
##  Mean   :2.5   Mean   :1
##  3rd Qu.:3.0   3rd Qu.:2
##  Max.   :3.0   Max.   :2
```

```
#Reassign variables types
switchglmsubo$Round = as.factor(switchglmsubo$Round)
switchglmsubo$Age = as.factor(switchglmsubo$Age)
switchglmsubo$Sex = as.factor(switchglmsubo$Sex)
switchglmsubo$Individual = as.factor(switchglmsubo$Individual)
switchglmsubo$Group.ID = as.factor(switchglmsubo$Group.ID)
switchglmsubo$Switches = as.numeric(switchglmsubo$Switches)

#Recheck status of variables
summary(switchglmsubo)
```

```
##     Group.ID    Individual Sex              Age      Round      Switches
##  1       : 2   3       : 2   S:32   Subordinate:32   2:16   Min.   :0
##  2       : 2   6       : 2                           3:16   1st Qu.:0
##  3       : 2   9       : 2                                  Median :1
##  4       : 2   12      : 2                                  Mean   :1
##  5       : 2   15      : 2                                  3rd Qu.:2
##  6       : 2   18      : 2                                  Max.   :2
##  (Other):20   (Other):20
```

```
#only subs to see if influence of round
#Set working directory
setwd("C:/Users/court/OneDrive/Desktop/OTI Binary Choice")

#Read glm.csv
switchglmsubo = read.csv("switch glm2r23sub.csv")


#Call packages
library(lme4)
```

```
## Loading required package: Matrix
```

```
library(lmerTest)
```

```
##
## Attaching package: 'lmerTest'

## The following object is masked from 'package:lme4':
##
##     lmer

## The following object is masked from 'package:stats':
##
##     step

library(car)

## Warning: package 'car' was built under R version 4.4.2

## Loading required package: carData

library(mclogit)
library(nnet)
library(memisc)

## Warning: package 'memisc' was built under R version 4.4.2

## Loading required package: lattice

## Loading required package: MASS

##
## Attaching package: 'memisc'

## The following object is masked from 'package:car':
##
##     recode

## The following object is masked from 'package:Matrix':
##
##     as.array

## The following objects are masked from 'package:stats':
##
##     contr.sum, contr.treatment, contrasts

## The following object is masked from 'package:base':
##
##     as.array

library(sjPlot)

## #refugeeswelcome

library(performance)

## Warning: package 'performance' was built under R version 4.4.2
```

```r
#Check status of variables
summary(switchglmsubo)
```

```
##     Group.ID       Individual        Sex                 Age
##  Min.   : 1.00   Min.   : 3.00   Length:32          Length:32
##  1st Qu.: 4.75   1st Qu.:14.25   Class :character   Class :character
##  Median : 8.50   Median :25.50   Mode  :character   Mode  :character
##  Mean   : 8.50   Mean   :25.50
##  3rd Qu.:12.25   3rd Qu.:36.75
##  Max.   :16.00   Max.   :48.00
##      Round         Switches
##  Min.   :2.0   Min.   :0
##  1st Qu.:2.0   1st Qu.:0
##  Median :2.5   Median :1
##  Mean   :2.5   Mean   :1
##  3rd Qu.:3.0   3rd Qu.:2
##  Max.   :3.0   Max.   :2
```

```r
#Reassign variables types
switchglmsubo$Round = as.factor(switchglmsubo$Round)
switchglmsubo$Age = as.factor(switchglmsubo$Age)
switchglmsubo$Sex = as.factor(switchglmsubo$Sex)
switchglmsubo$Individual = as.factor(switchglmsubo$Individual)
switchglmsubo$Group.ID = as.factor(switchglmsubo$Group.ID)
switchglmsubo$Switches = as.numeric(switchglmsubo$Switches)

#Recheck status of variables
summary(switchglmsubo)
```

```
##     Group.ID    Individual Sex              Age      Round     Switches
##  1      : 2   3      : 2   S:32   Subordinate:32   2:16   Min.   :0
##  2      : 2   6      : 2                           3:16   1st Qu.:0
##  3      : 2   9      : 2                                  Median :1
##  4      : 2   12     : 2                                  Mean   :1
##  5      : 2   15     : 2                                  3rd Qu.:2
##  6      : 2   18     : 2                                  Max.   :2
##  (Other):20   (Other):20
```

```r
#GLMER for number of switches (Frequency per trial round)
model3 = glm(Switches ~ Round , data = switchglmsubo, family = poisson)

summary(model3)
```

```
##
## Call:
## glm(formula = Switches ~ Round, family = poisson, data = switchglmsubo)
##
## Coefficients:
##             Estimate Std. Error z value Pr(>|z|)
## (Intercept)  0.06062    0.24254   0.250    0.803
## Round3      -0.12516    0.35425  -0.353    0.724
```

```
##
## (Dispersion parameter for poisson family taken to be 1)
##
##     Null deviance: 27.726  on 31  degrees of freedom
## Residual deviance: 27.601  on 30  degrees of freedom
## AIC: 81.738
##
## Number of Fisher Scoring iterations: 5
```

```r
anova(model3)
```

```
## Analysis of Deviance Table
##
## Model: poisson, link: log
##
## Response: Switches
##
## Terms added sequentially (first to last)
##
##
##       Df Deviance Resid. Df Resid. Dev Pr(>Chi)
## NULL                     31     27.726
## Round  1  0.12508        30     27.601   0.7236
```

```r
Anova(model3, type = "III")
```

```
## Analysis of Deviance Table (Type III tests)
##
## Response: Switches
##       LR Chisq Df Pr(>Chisq)
## Round  0.12508  1     0.7236
```

```r
tab_model(model3)
```

	**Switches**		
Predictors	*Incidence Rate Ratios*	*CI*	*p*
(Intercept)	1.06	0.63 – 1.65	0.803
Round [3]	0.88	0.44 – 1.77	0.724
Observations	32		
R^2 Nagelkerke	0.007		
