# Supplementary material for: Complex Residences and Sociality: How Coral Structure and Social Environment Influence Occupation Patterns in Gobiodon in Aquaria
Source: Ecol Evol. 2025 Jul 28;15(8):e71887. doi: 10.1002/ece3.71887 (PMC12304431; doi:10.1002/ece3.71887)
Supplement: Supplementary file 1 — Data S1: ece371887‐sup‐0001‐DataS1.zip. [file ECE3-15-e71887-s001.zip › ece371887-sup-0010-Appendix.docx]

## APPENDIX

Appendix Table 1 – Measurements used for the design and construction of coral models by TRICEP.

| **Complexity Level** | **Diameter (x axis cm)** | **Height (y axis cm)** | **Interbranch Distance (IBD) (cm)** | **Widest Branch Diameter (cm)** | **Thinnest Branch Depth (cm)** |
| --- | --- | --- | --- | --- | --- |
| **High** | 40 | 23 | 1.5 | 2.6 | 1.3 |
| **Low** | 40 | 23 | 3.2 | 2.6 | 1.3 |


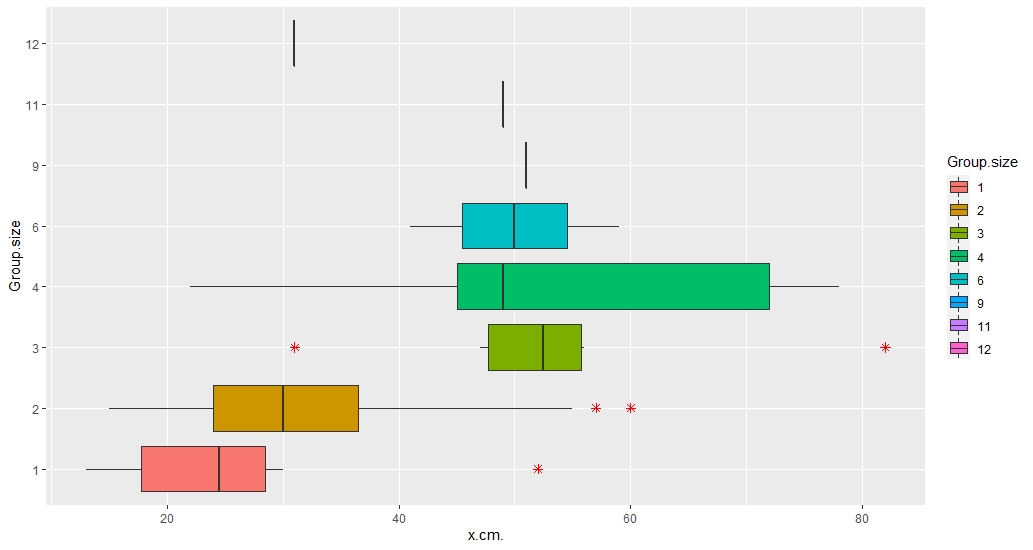


Appendix Figure 1 – Background data collected on a prior One Tree Island research trip in 2019 to survey *Gobiodon* populations. The boxplot displays range of combined diameter (x.cm) found from combined width, length and depth measurements of the corals hosted by groups of gobies. This information was utilised to obtain the dimensions for the 3D printed coral structures produced in collaboration with TRICEP.


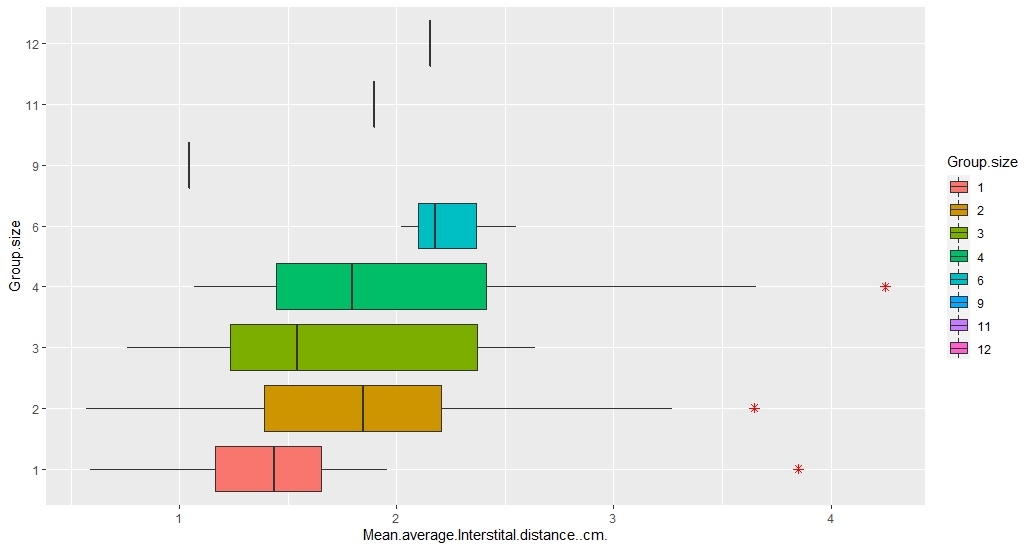


Appendix Figure 2 – Background data collected on a prior One Tree Island research trip in 2019 to survey *Gobiodon* populations. The boxplot displays the range of mean interstitial distance (cm) between branches for the corals hosted by groups of gobies. This information was used to obtain the dimensions for the 3D printed coral structures produced in collaboration with TRICEP.
